# Maternal metabolic factors and the association with gestational diabetes: A systematic review and meta‐analysis

**DOI:** 10.1002/dmrr.3532

**Published:** 2022-04-25

**Authors:** Nahal Habibi, Aya Mousa, Chau Thien Tay, Mahnaz Bahri Khomami, Rhiannon K. Patten, Prabha H. Andraweera, Molla Wassie, Jared Vandersluys, Ali Aflatounian, Tina Bianco‐Miotto, Shao J. Zhou, Jessica A. Grieger

**Affiliations:** ^1^ Robinson Research Institute University of Adelaide Adelaide South Australia Australia; ^2^ Adelaide Medical School University of Adelaide Adelaide South Australia Australia; ^3^ Monash Centre for Health Research and Implementation, School of Public Health and Preventive Medicine, Monash University Melbourne Victoria Australia; ^4^ Institute for Health and Sport Victoria University Melbourne Victoria Australia; ^5^ Department of Cardiology, Lyell McEwin Hospital Elizabeth Vale South Australia Australia; ^6^ School of Agriculture, Food and Wine, and Waite Research Institute, University of Adelaide Adelaide South Australia Australia; ^7^ School of Women's and Children's Health, University of New South Wales Sydney New South Wales Australia

**Keywords:** body mass index, gestational diabetes, glucose, lipids, meta‐analysis, metabolic syndrome, pregnancy

## Abstract

Gestational diabetes (GDM) is associated with several adverse outcomes for the mother and child. Higher levels of individual lipids are associated with risk of GDM and metabolic syndrome (MetS), a clustering of risk factors also increases risk for GDM. Metabolic factors can be modified by diet and lifestyle. This review comprehensively evaluates the association between MetS and its components, measured in early pregnancy, and risk for GDM. Databases (Cumulative Index to Nursing and Allied Health Literature, PubMed, Embase, and Cochrane Library) were searched from inception to 5 May 2021. Eligible studies included ≥1 metabolic factor (waist circumference, blood pressure, fasting plasma glucose (FPG), triglycerides, and high‐density lipoprotein cholesterol), measured at <16 weeks' gestation. At least two authors independently screened potentially eligible studies. Heterogeneity was quantified using *I* ^2^. Data were pooled by random‐effects models and expressed as odds ratio and 95% confidence intervals (CIs). Of 7213 articles identified, 40 unique articles were included in meta‐analysis. In analyses adjusting for maternal age and body mass index, GDM was increased with increasing FPG (odds ratios [OR] 1.92; 95% CI 1.39–2.64, *k* = 7 studies) or having MetS (OR 2.52; 1.65, 3.84, *k* = 3). Women with overweight (OR 2.17; 95% CI 1.89, 2.50, *k* = 12) or obesity (OR 4.34; 95% CI 2.79–6.74, *k* = 9) also were at increased risk for GDM. Early pregnancy assessment of glucose or the MetS, offers a potential opportunity to detect and treat individual risk factors as an approach towards GDM prevention; weight loss for pregnant women with overweight or obesity is not recommended.

Systematic review registration: PROSPERO CRD42020199225.

**Abbreviations**
BMI: body mass index
CVD: cardiovascular disease
DBP: diastolic blood pressure
GDM: gestational diabetes
HbA1c: glycated haemoglobin
SBP: systolic blood pressure

## 1 INTRODUCTION

Gestational diabetes mellitus (GDM) is defined as the onset or first recognition of glucose intolerance during pregnancy, primarily in the second or third trimester.^1^ GDM is one of the most common metabolic complications in pregnancy, affecting 5%–25% of all pregnant women worldwide, depending on screening approaches and diagnostic criteria.^2^ GDM has adverse maternal health consequences, including an increased risk for hypertensive disorders of pregnancy, preterm delivery, medicalised delivery,^3^, ^4^ as well as an increased risk for developing type 2 DM and cardiovascular events in the first decade following pregnancy.^5^, ^6^ Offspring of mothers with GDM are at greater risk for large for gestational age,^7^, ^8^, ^9^ respiratory distress syndrome^10^ and neonatal hypoglycaemia,^11^ and tend to develop type 2 diabetes at younger ages.^12^

Recognised risk factors for GDM include maternal obesity, advanced maternal age, excess gestational weight gain, Asian and African ethnicity, and a history of diabetes.^13^, ^14^, ^15^, ^16^, ^17^ Fasting or postprandial blood glucose may be assessed early, but whether it is a suitable screening test for GDM has not been clarified.^18^, ^19^, ^20^

Metabolic syndrome is a clustering of cardiovascular risk factors that includes atherogenic dyslipidemia, raised blood pressure, insulin resistance, and obesity,^21^ increasing the risk of cardiovascular disease (CVD)^22^ and diabetes by up to 5‐fold.^23^ In two pregnancy cohorts, Grieger et al.^24^ and Schneider et al.^25^ showed that MetS, measured in early pregnancy, increased the risk for GDM by 2–4 fold, even after adjusting for body mass index (BMI). Several studies have demonstrated that individual metabolic markers such as raised triglycerides (TG) or low density lipoprotein cholesterol, or reduced high density lipoprotein cholesterol (HDL‐C) pose a significant risk for developing GDM.^26^, ^27^, ^28^ The relationship between MetS or its individual components as a risk factor for GDM is plausible given their shared relationship to future risk of CVD. Importantly, metabolic factors can be modified by diet, lifestyle^29^, ^30^ and pharmacological agents.^31^ Consideration of assessing metabolic markers in early antenatal care may provide information about potential future risk for GDM, allowing for early detection and management.

While some systematic reviews have been conducted on similar topics, they did not specifically examine MetS factors, but rather explored biomarkers associated with placental pathology,^32^ central obesity,^33^ and predictive^34^ or diagnostic biomarkers for metabolic diseases.^35^ To date, there has been no systematic review or meta‐analysis comprehensively evaluating whether MetS or its components, measured in early pregnancy, associate with risk for GDM. This would be important given the current controversies surrounding early screening of GDM using conventional risk factors, and that intervention studies aimed at preventing GDM, predominantly through targeting hyperglycemia, have not been consistently successful.^36^ Measurement of MetS or its components may offer a new approach to identify potential risk for GDM, and which could be used as a complementary component to standard routine antenatal care.

The aim of this systematic review and meta‐analysis is to comprehensively evaluate the association between MetS and its components, measured in early pregnancy, and risk for GDM.

## 2 MATERIALS AND METHODS

We performed a systematic review and meta‐analysis of epidemiological studies examining the association between components of MetS and risk of GDM. The review was performed according to the PRISMA 2020 Guidelines (Preferred Reporting Items For Systematic Reviews and Meta‐analyses).^37^ The study protocol was registered on PROSPERO (International Prospective Register of Systematic Reviews) under the identification code: CRD42020199225 and is available online (www.crd.york.ac.uk/prospero).

### 2.1 Selection criteria and search strategy

Potential studies were identified through electronic database searches on Cumulative Index to Nursing and Allied Health Literature, PubMed, Embase, and the Cochrane database, and manual searches of potentially eligible references in review articles. The search strategy included a combination of subject indexing terms (i.e., MeSH) and free text search terms relating to early pregnancy, prognostic factors, and GDM, along with search filters recommended for prognostic modelling.^38^ The search strategy was iteratively developed by Jessica A Grieger and Nahal Habibi in consult with an academic librarian. The last search was performed on 5 May 2021. The full search strategy is provided in the Supporting Information.

The PICOTS criteria was used to define the aim, search strategy, inclusion and exclusion criteria, that is Population (Pregnant women), Index (Components of the MetS and MetS as a cluster), Comparator (Unexposed group [non GDM women]), Outcome (GDM measured at 24–28 weeks' gestation), Timing (recruitment <16 weeks' gestation), Setting (Antenatal care). The index prognostic factors included the following MetS factors: waist circumference (WC; abdominal obesity), systolic/diastolic blood pressure, glucose, glycated haemoglobin (HbA1c), TG, or HDL‐C, and MetS as a cluster.^39^, ^40^, ^41^ BMI was also examined because the International Diabetes Federation criteria for MetS includes BMI as a surrogate measure for WC. Excluded studies were those examining diagnostic models; animal models; pregnancy outcomes in women after GDM diagnosis; or studies assessing measurements that are not assessed routinely in antenatal care (e.g., using bioelectrical impedance assay).

#### 2.1.1 Core outcomes

The primary outcome was GDM using any diagnostic criteria, measured at 24–28 weeks' gestation.

### 2.2 Study selection, data collection and risk of bias assessments

All citations were imported into an Endnote file, duplicates were removed, and the remaining articles export into the Rayyan software database for blind screening.^42^ Title and abstract screening was completed in duplicate by two authors independently (Jessica A. Grieger, Prabha H. Andraweera, Molla Wassie, Mahnaz Bahri Khomami, Tina Bianco‐Miotto, Jared Vandersluys, Shao J Zhou, Nahal Habibi, Aya Mousa), and any disagreements were resolved by consensus between the two authors. Where necessary, authors of included articles were contacted to provide missing information and/or unpublished data.

Data extraction was performed by at least two authors independently (Nahal Habibi, Rhiannon K. Patten, Aya Mousa, Chau Thien Tay, Prabha H. Andraweera, Mahnaz Bahri Khomami, Molla Wassie, Jared Vandersluys, Ali Aflatounian), using a specifically designed Microsoft Excel spreadsheet. Cross‐checking and resolving of differences were completed by Jessica A. Grieger. Data extraction was guided by CHARMS‐PF (Critical Appraisal and Data Extraction for Systematic Reviews of Prediction Modelling Studies): a checklist of key items to be extracted from primary studies of prognostic factors.^43^ Data extraction included: author, year, country; type of study; study population and sample size; study duration and month/year the study was carried out; inclusion criteria; exclusion criteria; GDM diagnosis and time point; exposures in the model; and statistical adjustments. When risk estimates from more than one multivariable analysis were reported, data were extracted from the analysis adjusting for the largest number of confounders. If risk estimates from other routine antenatal factors were reported, only risk estimates related to MetS were extracted. Only standard cut‐off values for categorical data were used, for example, the World Health Organization (WHO) categories for BMI. Outcome data reported as odds ratios (OR)/relative risks (RR) and 95% confidence intervals (CIs) were the primary output of interest.

Risk of bias assessment at the study‐level was performed independently by two researchers, using the quality in prognostic factor studies (QUIPS) Risk of Bias tool.^43^ The same two independent authors who completed the data extraction completed the risk of bias for the same set of studies. Domains included study participation; study attrition; prognostic factor measurement; outcome measurement; adjustment for other key prognostic factors; and statistical analysis and reporting. Each domain was judged as low, moderate or high risk, with more weighting given to the domains of ‘Adjustment for other prognostic factors’ and ‘Statistical analysis and reporting’, due to the observational nature of the included studies. Pilot testing was performed using three test articles to ensure consistency between the authors prior to formally commencing risk of bias assessments. For each study, the item scores were collated and an overall risk of bias (low, moderate, and high) was determined.

### 2.3 Data analysis

All analyses were conducted using Review Manager (V5.4.1). For the primary meta‐analyses, studies reporting OR or RR with 95% CIs were analysed as these data are best suited to address questions on prognosis. Data were pooled using the restricted maximum likelihood random‐effects models to account for heterogeneity among the studies and outcome measures.^44^ Unadjusted analyses were firstly reported followed by adjusted analyses with a core set of prognostic covariates (maternal age, maternal BMI, family history of diabetes, ethnicity). As many of the included studies did not adjust for all four covariates, we opted for at least one core covariate in each model.

Heterogeneity was quantified using the *I* ^2^ statistic. Significance for heterogeneity was set at *p* < 0.10, with an *I* ^2^ > 50% considered to be of relatively high heterogeneity.^45^ Sources of heterogeneity were explored where outlier study/s were eliminated from the meta‐analysis in a series of sensitivity analyses and the effect size was recalculated to determine the influence of those studies.^46^ We considered outlier studies that had a different direction of effect, a high effect size, or studies judged to be at high risk of bias.^45^ If ≥10 studies were available, we assessed publication bias by visual inspection of funnel plots.^47^ Data reported as mean and SD/SE or 95% CI, or as median and interquartile range were included in the narrative synthesis.

### 2.4 Patient and public involvement

This study was a systematic review and therefore did not include patients as study participants.

## 3 RESULTS

### 3.1 Study selection and characteristics

**FIGURE 1:**
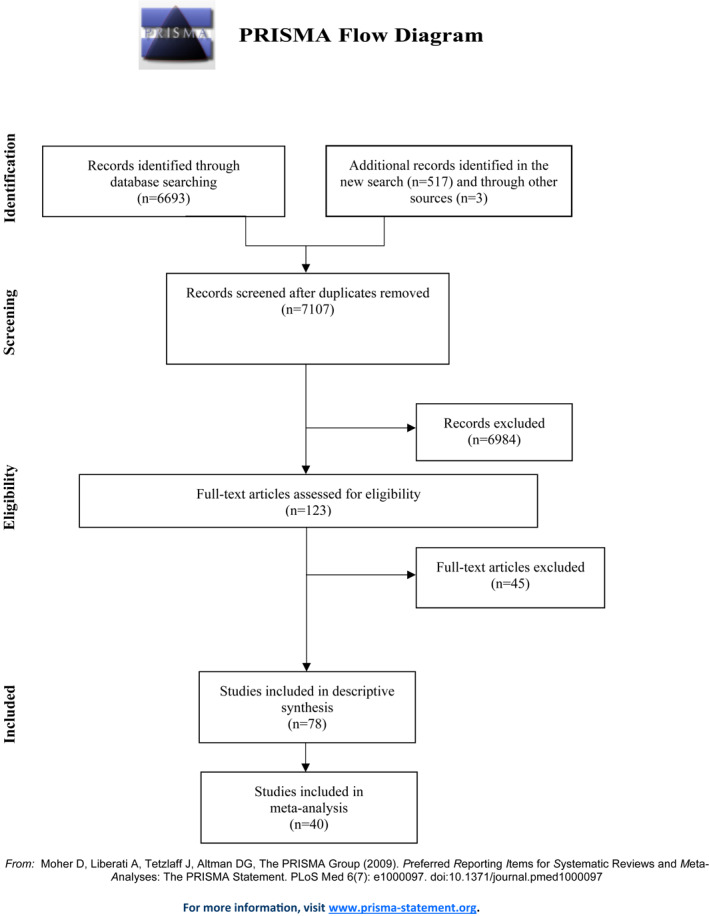
PRISMA flow diagram

The systematic search identified 7213 articles of which 106 were duplicates, 7029 were ineligible, leaving 78 articles in the systematic review and 40 articles in the meta‐analysis (Figure 1). Characteristics of the included studies are reported in Table 1. The majority of studies were conducted in China (*k* = 18 studies), USA (*k* = 9 studies), UK (*k* = 8 studies), and Australia (*k* = 8). Study population sizes ranged from 107^48^ to 132,899 participants.^49^ Of the 78 studies included, the majority (41.25%) reported on two or more MetS factors. Body mass index was the most common independently assessed risk factor (64.1%), while 10 studies (12.8%) reported on HbA1c and HDL‐C.

**TABLE 1:** Characteristics of the studies included in the systematic review

Author, country	Type of study	Study population and sample size	Years	Inclusion criteria	Exclusion criteria	GDM diagnosis and time point	Main exposure in model (e.g., BMI, TG)	Adjustments made
Madhavan 2008, India	Prospective cohort	Outpatients of Ob/Gyn Department, Government Medical College Hospital, Kottayam, Kerala. Enroled during first antenatal visit *n* = 106; GDM *n* = 8	April 2005–April 2006	Single live intrauterine pregnancies; gestational age ≦12 weeks' gestation; maternal age 18–35 years	History of diabetes pre‐pregnancy, history of drugs known to cause insulin resistance within prior 6 months; history of thyroid or pituitary disorders; comorbid conditions and severe systemic illness	World Health Organization (WHO) 1999 criteria, at 24–28 weeks' gestation.	BMI, waist circumference (WC), waist hip ratio (WHR) and fasting blood sugar	Not reported
Cozzolino 2017, Italy	Single centre retrospective cohort study	*n* = 656	January 2010–January 2016	All multiple pregnancies screened for GDM with 75 g, 2 h OGTT at 24–28 weeks' gestation	Maternal pre‐gestational diabetes and hypertension or other chronic diseases (i.e., cardiovascular, autoimmune diseases, inherited and acquired thrombophilia); absence of the 75 g OGTT screening during pregnancy; major foetal congenital anomalies; twin‐to‐twin transfusion syndrome; miscarriage or intrauterine foetal death before the OGTT	International Association of Diabetes and Pregnancy Study Groups criteria (IADPSG), at 24–28 weeks' gestation	BMI	Not reported
Hinkle 2018, USA	Nested case control study	Enroled between 8 and 13 weeks' gestation; *n* = 2802; GDM *n* = 107, matched non‐GDM controls *n* = 214	2009–2013	Low‐risk, pregnancies among non‐obese women and 468 pregnancies among obese women (*n* = 2802 total). The inclusion criteria for obese cohort included women who smoked prior to pregnancy, had a haematologic disorder, or had GDM in prior pregnancy.	Non‐obese women who smoked, had GDM in a prior pregnancy or had a haematologic disorder (e.g., chronic anaemia, sickle cell disease, low platelets, blood clotting problems) were excluded; as were women with HbA1c ≥ 6.5% (48 mmol/mol) at enrolment (*n* = 3) or who had a haemoglobin variant (*n* = 6).	Carpenter and Coustan criteria, as endorsed by the American Diabetes Association (ADA), and the American College of Obstetrics and Gynaecologists (ACOG), at 24–29 weeks' gestation.	HbA1c	Maternal age, gestational age at delivery, family history of diabetes, pre‐pregnancy overweight and obesity
Zhu 2020, China	Prospective cohort	*n* = 2949; GDM *n* = 581 (19.7%)	July 2016–June 2017	Between 6 and 8 weeks' gestation; aged >18 years; singleton pregnancy; regular prenatal visits; opted to deliver at Fu Xing Hospital	Pre‐pregnancy cardiovascular disease; chronic hypertension; pre‐pregnancy diabetes; thyroid disorder; taking medications known to affect glycaemic and lipid metabolism; twin pregnancy	IADPSG criteria, at 24–28 weeks' gestation	Fasting (>8 h) triglycerides (TG) at 6–8 weeks' gestation; stratified for pre‐pregnancy BMI	Maternal age, fasting blood glucose, pre‐pregnancy BMI, family history of DM
Godwin 1999, Canada	Retrospective cohort	*n* = 1298; GDM *n* = 110 (8.5%)	1 January 1987–31 December 1995	Swampy Cree women who gave birth at Weeneebayko Hospital, Moose Factory, James Bay, Ont.	Women who were transferred to other hospitals (*n* = 30). Large amounts of data missing	International Workshop‐Conference on Gestational Diabetes; or fasting sugar or a 1‐h 50‐g challenge test was done, resulting in a blood glucose value of ≧7.8 mmol/L. GDM diagnosis time‐point not reported.	Diastolic blood pressure, weight at first visit	Age, history of GDM in a previous pregnancy, diastolic blood pressure, weight at first prenatal visit, first degree relative with GDM
Grieger 2018, Australia and New Zealand	Prospective cohort	*n* = 3126 (55.6% of total population); GDM = 14.7%	November 2004 – November 2011	Low‐risk, nulliparous women, at 14–16 weeks' gestation with singleton pregnancies recruited from Adelaide (Australia), Auckland (New Zealand), Cork (Ireland), Leeds (UK), London (UK), and Manchester (UK).	High risk for various pregnancy complications, including preeclampsia, small for gestational age or spontaneous preterm birth; took high dose vitamin supplements; type 1 or 2 diabetes	WHO 2013 criteria, at 24–28 weeks' gestation.	Waist circumference (WC), TG, HDL‐C, sBP, dBP, glucose, MetS	Maternal BMI, age, study centre, SEI, ethnicity, foetal sex, physical activity, smoking status, depression status
Doi 2020, UK	Retrospective cohort	*n* = 132,899; GDM *n* = 1877 (1.42%)	January 2007 – December 2015	All women within the Scottish Morbidity Record 01 or 02 or Scottish Birth Record with first time singleton deliveries.	Mothers aged below 20 years and over 40 years were excluded (post hoc)	WHO's International Classification of Diseases, Tenth revision (ICD‐10). GDM diagnosis time‐point not reported.	BMI	Maternal age at delivery, smoking during pregnancy, Carstairs 2001 quintiles for socioeconomic status in Scotland
Yachi 2011, Japan	Prospective cohort	Pregnant women who visited the obstetrics clinic in Tokyo <13 weeks' gestation, *n* = 509	September 2008–January 2010	Pregnant women who visited the obstetrics clinic in Tokyo <13 weeks' gestation and without recognised diabetes prior to pregnancy	Fasting plasma glucose (FPG) levels <2.5 mmol/l (*n* = 3). Missing or incomplete blood glucose data (*n* = 15).	Japan Society of Obstetrics and Gynaecology criteria, at 24 –29 weeks' gestation.	BMI, FPG	Maternal age, parity, BMI at first prenatal visit, gestational weight gained per week up to GCT.
Iyoke 2013, Nigeria	Nested case control study within a retrospective cohort	All booked parturient women who delivered at three major maternity centres; *n* = 648; early pregnancy obesity *n* = 324; control (normal weight) *n* = 324	1 January 2010–31 December 2011	Cases: Parturient women with BMI ≥30 kg/m^2^	*n* = 16 obese women refused to be included in the study. Only included obese women and matched controls.	Not reported.	BMI	Not reported
				Controls: Parturient women who booked in the first trimester with normal BMI and matched with cases in age and parity.				
Schrauwers 2009, Australia	Retrospective cohort	Singleton pregnancies at the Lyell McEwin Hospital, South Australia *n* = 370	January 2006–June 2006	Singleton pregnancies delivered at Lyell McEwin Hospital, Adelaide with complete medical records.	Not reported	Case records; no other data reported.	BMI	Not reported
Migda 2016, Poland	Prospective observational	Cases: Caucasian women in singleton pregnancies (*n* = 124) between 11 and 13 weeks' gestation with metabolic syndrome (MetS).	2011–2013	Single, live pregnancy and 3 of 5 risk factors: Population‐specific elevated WC; drug treatment for elevated TG or elevated blood pressure or elevated fasting glucose; reduced high density lipoprotein cholesterol (HDL‐C) <40 mg/dl (1.0 mmol/L) in males and <50 mg/dl (1.3 mmol/L) in females. Controls: 30 women with healthy pregnancies.	Not specified but they included 124 cases from the total of 127 cases.	Polish Gynaecology Society criteria, at 24–28 weeks' gestation	MetS	Not reported
		Controls (*n* = 30): Healthy pregnant women						
		GDM, *n* = 19						
Kouhkan 2018, Iran	Nested case‐control	Singleton pregnancies *n* = 270; GDM *n* = 135, controls *n* = 135	October 2016–June 2017	ART singleton pregnancy, aged 20–40 years	Pre‐existing diabetes; multiple pregnancy and chronic diseases such as hypertension, cardiovascular diseases, untreated thyroid disease, liver diseases, renal diseases, autoimmune diseases, and connective tissue disorders; those taking corticosteroids	ADA/IAPDSG criteria, at 24–28 weeks' gestation	Fasting blood sugar, blood pressure	Age and BMI, family history of diabetes, and gravidity
Phaloprakarn 2009, Thailand	Retrospective cohort	Cohort 1 *n* = 1876; GDM *n* = 586 (31.2%) Cohort 2 (validation cohort) *n* = 1900; GDM *n* = 469 (24.7%)	Cohort 1 March 2005–October 2006. Cohort 2 July 2007–December 2005	Both cohorts; singleton pregnancy, no overt diabetes, certain last menstrual period (LMP), first trimester (14 weeks' gestation)	Not reported	Carpenter and Coustan criteria, at 24–28 weeks' gestation	BMI	Age, parity, family history of diabetes, prior macrosomia, history of ≥2 abortions
Sweeting 2017, Australia	Retrospective case control	*n* = 978; GDM *n* = 248, non‐GDM *n* = 730	April 2011–May 2013	Singleton pregnancy; attending Royal Prince Alfred Hospital, Sydney; at 11 –13 + 6 weeks' gestation	Pre‐existing diabetes; pre‐eclampsia; multiple pregnancies; pre‐term delivery (<37 weeks' gestation); miscarriage; stillbirth; termination; foetal chromosomal abnormality; missing clinical data; where GDM was diagnosed based on a glucose challenge test alone	Australasian Diabetes in Pregnancy (ADIPS) diagnostic criteria, at 24–28 weeks' gestation	BMI	Previous GDM, family history of diabetes, age, south/east Asian ethnicity, parity.
Gur 2014, Turkey	Prospective cohort	*n* = 106; included *n* = 94	January 2012–January 2013	Maternal age 18–40 years; singleton 4–14 weeks' gestation	Pregnant subjects with type 1 or 2 diabetes; hypertension; any additional metabolic disease; on chronic drug therapy; newly diagnosed type 2 diabetes based on GCT and OGTT during the study (*n* = 6); lost to follow up (*n* = 6)	National Diabetes Group criteria, at 24 weeks' gestation.	WC, BMI, BP, glucose, total cholesterol (TC), TG, HDL, LDL, insulin, homoeostasis model assessment‐insulin resistance index (HOMA‐IR)	Maximum pre‐peritoneal visceral fat, minimum subcutaneous fat and BMI
Lei 2016, China	Prospective cohort	*n* = 5535; GDM *n* = 1138 (20.56%)	January 2012–December 2014	If they attended before 20 weeks' gestation (mean 16 weeks)	Multiple pregnancy, conception by means of gonadotropin ovulation induction or in vitro fertilisation, ischaemic heart disease, stroke, peripheral vascular disease, dyslipidemia, diagnosis of diabetes or/and hypertension before current pregnancy	IADPSG criteria, at 24–28 weeks' gestation.	BMI, fasting plasma glucose, HDL‐C and TG, blood pressure	Maternal age and parity
Zhu 2019, USA	Prospective cohort study followed by a nested case‐ control	*n* = 1839; Screened GDM	Pregnancies delivered as of August 2016	Multi‐racial/ethnic pregnant women, aged 18–45 years, <11 weeks' gestation	Multiple gestations, pre‐existing diabetes, cancer, hepatitis C, liver cirrhosis, pregnancy termination, diagnosis of diabetes/use of diabetes medication before baseline examination, missing WC or HC data (*n* = 9)	Carpenter and Coustan criteria, at 24–28 weeks' gestation	BMI, WC	Risk estimates were adjusted for gestational age at waist and hip circumference measurement
		*n* = 1759; final cohort sample *n* = 1750, GDM *n* = 186 (10.6%) nested case‐control study: GDM *n* = 115, matched controls *n* = 230						
Zhu 2013, China	Prospective	*n* = 17,186; GDM *n* = 3002 (17.5%)	January 1 – 29 February 2012	All women registered to clinic during those times whose blood glucose test results were linked to gestational week.	Pre‐existing diabetes	Criteria established by Ministry of Health China. Diagnosis with meeting or exceeded 75 g OGTT: 0 h (fasting), 5.10 mmol/L; 1 h, 10.00 mmol/L; and 2 h, 8.50 mmol/L. 24–28 weeks' gestation	FPG	Not reported
Zhang 2019, China	Prospective observational	*n* = 1704; GDM *n* = 544 (37.2%)	March 2017–September 2017	Healthy women; natural conception; singleton pregnancy; gestational age 8–12 weeks' gestation	Type 1 and 2 diabetes prior to pregnancy; fasting plasma glucose >7 in 1st trimester; cardiovascular diseases; inherited metabolic diseases or thyroid diseases	IADPSG criteria, at.24–28 weeks' gestation	TG, HDL‐C (also TC and LDL‐C)	Maternal age, pre‐pregnancy BMI, gravidity, parity, history of GDM, family history of DM, maternal education, family income, exercise habits pre‐pregnancy, exposure to passive smoking before/during pregnancy, energy intake and expenditure.
Magann 2013, Australia	Retrospective cohort study	*n* = 4490; GDM BMI < 25 kg/m^2^ *n* = 2.8%	January 2007–July 2008	Initial antenatal visit in 1st trimester; singleton pregnancies >20 weeks' gestation	Not reported	Not reported	BMI	Maternal age, nulliparity, ethnicity, pre‐existing diabetes, pre‐existing hypertension, pregnancy weight gain
Zhao 2014, China	Retrospective cohort	*n* = 411; GDM *n* = 52 (12.7%)	2010–2011	Not reported	Not reported	Not reported	BMI, TG	Maternal age
Simko 2019, Slovakia	Retrospective cohort	*n* = 7122; maternal underweight *n* = 741 (10.4%), normal weight *n* = 5400 (76.0%), overweight *n* = 602 (8.5%), obese *n* = 358 (5.0%)	1 January 2013–31 December 2015	Singleton deliveries >37 weeks' gestation	Pregnancies with chronic hypertension; foetal anomalies; type 1 and 2 diabetes	50 g OGTT at 24–28 weeks' gestation: Fasting and 2 h post 75 g OGTT values were >5.5 mmol/I and >8 mmol/l, respectively. No other criteria/guideline reported.	BMI	Maternal age, gestational age, gestational weight gain, smoking
O'Malley 2020, Ireland	Prospective observational cohort	Women with at least one maternal risk factor for GDM *n* = 202; GDM *n* = 108 (53.5%)	October 2017‐ November 2018	Maternal age ≥18 years; understood English; ≥1 maternal risk factor for GDM	Multiple pregnancy; pre‐existing diabetes mellitus.	WHO 2013 criteria, at 26–28 weeks' gestation.	Obesity (no BMI reported), TG, HDL‐C (non‐MetS = TC, LDL‐C, TG:HDL‐C ratio)	Pre‐pregnancy BMI
Wang 2016, China	Retrospective cohort	*n* = 5218; GDM *n* = 1053 (20.2%)	20th June–30th November 2013	Live‐born singleton infant; full information on early pregnancy lipid profiles (14 weeks' gestation); pregnancy course and outcome	Pre‐existing diabetes; hypertension; thyroid disease or immune system disorders; multiple births; missing data on major items such as pre‐pregnancy weight, height, 75 g OGTT results, PE diagnosis, birth weight and gestational age	75 g OGTT >24 weeks' gestation. Diagnosis of GDM made when any one value met or exceeded the following values: 0 h, 5.1 mmol/L; 1 h, 10.0 mmol/L; 2 h, 8.5 mmol/L.	TG, HDL‐C (also TC and LDL‐C)	Maternal age, pre‐pregnancy BMI, gravidity, parity, education, family history of diabetes, gestational age at time of lipid measurement. Used to estimate ORs for the associations between GDM and early pregnancy lipid levels.
El‐Gilany 2010, Saudi Arabia	Prospective cohort	*n* = 787; GDM *n* = 30 (3.8%)	2007	All women attending PHCCs for antenatal care within the first month of pregnancy and willing to come for regular follow‐up throughout pregnancy	Any pre‐pregnancy chronic medical disease (e.g., hypertension, diabetes, renal or cardiac disease, sickle cell disease), multiple pregnancies	Not reported	BMI	Not reported
Wen‐Yuan 2016, China	Prospective population‐based cohort	Chinese women pregnant at 28–37 weeks' gestation; *n* = 934; GDM *n* = 71 (7.6%)	June 2010–June 2011	Pregnant at 28–37 weeks' gestation; integrated medical records and clear gestational age; singleton pregnancy; naturally conceived	Multiple pregnancy; diabetes; chromosomal abnormalities; inherited metabolic diseases or thyroid diseases before pregnancy; experienced serious infection during early pregnancy; conceived with assisted reproductive techniques	IADPSG criteria, at 24–28 weeks' gestation	Fasting bloods taken at 7–10 weeks' gestation for TC, TG, HDL‐C and LDL‐C concentrations, maternal pre‐pregnancy BMI (WHO categories (41))	Maternal age, pre‐pregnancy BMI, gestational weight gain, parity, maternal education, socioeconomic status, infant sex and delivery mode, family income, smoking
Denison 2014, UK	Retrospective population‐based cohort	<16 weeks' gestation recruited *n* = 109,592 (124,280 deliveries); GDM *n* = 503 (4.4%).	January 2003–February 2010	Maternal BMI recorded <16 weeks' gestation; weight 35–140 kg	>140 kg, >44 weeks' gestation, birth weight >6 kg, BMI assessed >16 weeks' gestation	Scottish morbidity records 2 (SMR02) held at ISD of NHS Scotland. No time point recorded.	Maternal BMI <16 weeks' gestation, grouped according to WHO BMI categories (41).	Maternal age, smoking, Carstrairs quintile
Han 2018, China	Prospective population‐based cohort	*n* = 17,803; GDM *n* = 1383 (7.8%)	October 2010–August 2012	Women registered with primary care hospital at <12 weeks; non‐fasting 50 g 1 h GCT at 24–28 weeks' gestation	Did not undergo GCT; positive GCT but did not undergo formal OGTT; had pre‐existing diabetes	IADPSG criteria, at 24–28 weeks' gestation.	BMI, WC	Maternal age, height, family history of DM in 1st degree relatives, GA at registration, parity ≥1, education >12 years, Han nationality, non‐singleton pregnancy, SBP at registration, weight gain per week from registration to GCT, smoking and drinking status before pregnancy, BMI, WC
Pazhohan 2019, Iran	Prospective	24–29 years 48.5% of study cohort was overweight or obese. *n* = 954; control *n* = 778, GDM *n* = 176	August 2014–February 2016	Singleton pregnancy at 1st trimester; attended health centres for first prenatal visit and invited to participate in the study; blood sampling at 9 weeks' gestation	Type 1 or 2 diabetes pre‐pregnancy; FPG ≥126 mg/dl in the first trimester of current pregnancy; cardiovascular diseases; maternal age 18–35 years	IADPSG criteria, at 24–28 weeks' gestation.	FPG, TC, HDL‐C, LDL‐C, TG, TG/HDL‐C ratio, LDL/HDL ratio, TyG index (TG glucose index)	Age, family history of diabetes, 1st trimester BMI
Syngelaki 2011, UK	Prospective	Singleton pregnancies at 11–13 weeks' gestation *n* = 45,191; included *n* = 41,577	Not reported	Singleton pregnancies with live foetus and crown rump length of 45–84 mm at 11–13 weeks' gestation, complete data.	Pregnancies conceived by intrauterine insemination incomplete data on pregnancy outcome, pre‐pregnancy type 1 or 2 diabetes, ending in miscarriage or delivery <30 weeks (no screening and diagnosis of GDM) and foetal death <24 weeks.	WHO criteria, 2006 at 24–28 weeks' gestation.	BMI	Maternal age, racial origin, method of conception, cigarette smoking during pregnancy, history of chronic hypertension, history of type 1 or 2 diabetes mellitus (DM), and obstetric history including the outcome of each previous pregnancy
Sánchez‐Vera 2007, Spain	Prospective nested case‐control	*n* = 107; GDM *n* = 62, non‐GDM *n* = 45	July 2001–July 2004	All women attending obstetric clinic asked to participate; only white women who spoke Spanish fluently included; blood tests performed during routine visits at 15, 24 and 32 weeks	Immigrant women not fluent in Spanish; type 1 and 2 diabetes; multiple pregnancy	American Diabetes Association (ADA), at 24 weeks' gestation	Glucose, TC, TG, weight, BMI	Plasma levels of cholesterol, TG, vitamin E, oestradiol, progesterone, obesity, time of gestation
Falcone 2019, Austria	Prospective cohort	*n* = 574; GDM *n* = 103, non‐GDM = 471	January 2016–July 2017	Not reported	Pre‐existing diabetes	IADPSG criteria at the late second or early third trimester (exact gestational week not reported)	Fasting HbA1c, plasma glucose, insulin, C‐peptide	Age and BMI
Sesmilo 2019, Spain	Retrospective analysis	*n* = 6845; GDM *n* = 695 (10.2%)	2008–2018	Patients with an available FPG in 1st trimester performed in the laboratory under standard conditions, result <110 mg/dl, patients who had complete data for all outcomes	Patients <18 years, pregestational diabetes, multiple pregnancies and/or pregnancies by means of in vitro fertilisation or gonadotropin ovulation induction	NDDG criteria, in 2nd trimester.	FPG	Multivariate logistic model adjusted by maternal age, BMI at the first antenatal visit, previous pregnancies, gestational age, weight gained in pregnancy (transformed into Z‐score) and tobacco use was fitted.
Amylidi 2016, Switzerland	Observational retrospective cohort	*n* = 208; GDM *n* = 32 (15.2%)	June 2011–November 2012	Pregnant women attending antenatal clinic with at least one of: BMI ≥ 30 kg/m^2^, first‐degree family member with diabetes, PCOS, ethnicity (African, Latino, Asian, Pacific Islander), previous pregnancy with GDM or delivery of an infant ≥4.5 kg.	Women with pre‐existing diabetes or a first‐trimester HbA1c ≥ 6.5% (≥48 mmol/mol)	ADA at 24–28 weeks' gestation.	HbA1c, BMI	Not reported
Vellamkondu 2017, India	Prospective observational	Women booked between 11 and 14 weeks *n* = 440; GDM *n* = 38	Over 2 years (not stated)	Pregnant women booked between 11 and 14 weeks, singleton viable pregnancy, chose to undergo combined screening for aneuploidy (including nuchal translucency and serum biochemistry)	Not reported	Not reported	BMI	Not reported
Wang 2013, China	Prospective	*n* = 738; PCOS *n* = 114, controls *n* = 594	January 2010–December 2012	Women diagnosed with PCOS (*n* = 220) and a matching control group (*n* = 652); pregnancy confirmed by transvaginal ultrasonography between 6 and 8 weeks' gestation	>40 years, pre‐existing diabetes, cardiomyopathy accompanied by cardiac insufficiency, active hepatitis, uncontrolled hyperthyroidism, active systemic lupus erythematosus, serious hematopathy, malignant tumours, serious trauma, smoking, drug/alcohol use, organic pelvic disease, pregnancy accompanied with acute abdominal disease	At least two values ≥: fasting glucose 5.1 mmol/L, 1 h level 10.0 mmol/L, and 2 h level 8.5 mmol/L. 24–28 weeks' gestation.	BMI	Incidence of pregnancy outcomes according to conception methods (spontaneous conception, IVF‐ET, or ovarian stimulation), age at conception (≤30 years or >30 years), BMI ((<24 kg/m^2^ (lean) or ≥24 kg/m^2^ (overweight/obesity)), glucose tolerance state (NGT or GDM)
Knight‐Agarwal 2016, Australia	Retrospective cohort	Women from a Birthing Outcome System database, 1st antenatal visit ∼12 weeks' gestation *n* = 14,857	January 2008–December 2013	Not reported	Women with missing BMI data and multiple pregnancies were excluded	International Classification of Diseases (ICD)‐10 codes and standard operating procedures developed by the tertiary institution where the study was conducted.	BMI	Maternal age, parity, country of birth, smoking status
Collier 2017, UK	Retrospective cohort	Data extracted from the Scottish Morbidity Record 02, >31 years *n* = 1,891,097; included in analysis (from 2012 subgroup) *n* = 47,290	1 January 1981–31 December 2012	Not reported	Delivering at home or in non‐NHS hospitals	Coded as GDM or if any of the diagnosis were coded as O244 (ICD10) or 6488 (ICD9) in the SMR02 dataset.	BMI	Maternal BMI, maternal age, parity status, smoking status, maternal SIMD status
Savvidou 2010, UK	Nested case‐control study	First trimester maternal samples from 124 women who developed GDM and 248 control subjects who did not (11 + 0–13 + 6 weeks)	Not stated	Not stated‐ All women had phenotypically normal neonates.	Women with pre‐existing diabetes and twin pregnancies were excluded	1999 WHO criteria, at 24–28 weeks' gestation	BMI, BP, TC, LDL, HDL, non‐fasting TG,	Maternal age, BMI, gestational age at sampling, smoking, ethnicity, parity, conception status, and previous GDM
Kansu‐Celik 2019, Turkey	Retrospective cohort	Women at 1st trimester screening between 6 and 14 weeks' gestation; *n* = 608; GDM *n* = 69, non‐GDM *n* = 539	January 2010–January 2018	HbA1c levels were measured <14 weeks' gestation	Multiple gestations, clinical evidence or history of any systemic disease or pregestational diabetes (types 1 and 2), hypertension, FPG exceeding 126 mg/dl, 2 h postprandial values exceed 200 mg/dl, HbA1c ≥ 6.5% during any gestational week, a GCT above 200 mg/dl between gestational weeks 24–28 weeks, positive OGTT during first trimester, history of kidney, liver, or thyroid disease.	Carpenter and Coustan criteria, at 24–28 weeks' gestation.	FPG, HbA1c	Not reported
Farah 2012, Ireland	Prospective observational	*n* = 2000; included *n* = 1935; GDM screening *n* = 547, GDM *n* = 70	July 2008–March 2010	White European women; singleton pregnancy	Pre‐pregnancy diabetes; maternal age <18 years; unable to give consent	ADA criteria, diagnosed around 28 weeks' gestation	BMI	Not reported
Bao 2018, USA	Nested case‐control	*n* = 321; GDM *n* = 107, non‐GDM *n* = 214	2009–2013	4 race/ethnic groups; maternal age 18–40 years; singleton pregnancy; pre‐pregnancy BMI 19–45 kg/m^2^	HIV; major chronic conditions such as pre‐ pregnancy hypertension, pre‐pregnancy diabetes, cancer, psychiatric, renal or autoimmune diseases	ACOG criteria. Diagnosis time point not reported (but excluded women with GDM <26 weeks' gestation)	TC, HDL, TG, LDL	Maternal age, gestational age at blood collection, parity, family history of diabetes, pre‐pregnancy BMI
Odsæter 2015, Norway	Prospective RCT post hoc analyses	*n* = 228; dropped out *n* = 12, GDM‐WHO *n* = 55 (24.1%), GDM‐IADPSG *n* = 35 (15.4%)	February 2995–January 2009	Pregnant women with PCOS, 18–45 years, singleton pregnancy between 5 and 12 weeks' gestation	ALT >90 IU/l; creatinine >130umol/l; known alcohol abuse, previous DM, fasting plasma/serum glucose >7.0 mmol/l at inclusion, treatment with glucocorticoids or use of drugs known to interfere with metformin	WHO 1999 criteria, at 24–28 weeks' gestation.	HbA1c	GDM 1st trimester and GDM throughout pregnancy: HbA1c, age and BMI at inclusion, GDM in previous pregnancy, using metformin at conception/early pregnancy pre‐eclampsia: HbA1c, age and BMI at inclusion, using metformin at conception or during pregnancy, GDM‐WHO in 1st trimester, nulliparity, smoking in 1st trimester, pre‐eclampsia in previous pregnancy, pre‐gestational HTN birth weight: HbA1c, age and BMI at inclusion, using metformin at conception or during pregnancy, GDM‐WHO in 1st trimester, nulliparity, smoking in 1st trimester
Yang 2019, Australia	Retrospective cohort	Singleton deliveries *n* = 35,099 (GDM data *n* = 24,161 (70% retained); GDM *n* = 2126 (8.8%), non‐GDM *n* = 22,034 (91.2%)	2009–2015	ACT residents, singleton birth, pregnancy duration of between 24 and 43 weeks	Missing maternal height or weight	Not reported (some self‐reported)	1st antenatal visit BMI	Maternal age, parity, smoking in pregnancy, Aboriginal and TSI status, socio‐economic indexes for areas
Sreedevi 2012, India	Observational study (unclear if prospective or retrospective)	*n* = 250; GDM *n* = 40, non‐GDM *n* = 210	Not reported	Women who registered between 7 and 10 weeks' gestation, regular antenatal check‐up and complete records of antenatal and intranatal periods	Not reported	Not reported	First trimester BMI	Not reported
Zheng 2019, China	Prospective cohort	Cohort 1 *n* = 566; PCOS *n* = 242, controls *n* = 324	Cohort 1 January 2013–December 2015.	8–15 weeks' gestation; singleton pregnancy; maternal age 18–45 years; history of PCOS (or age and pre‐pregnancy BMI matched controls)	Pre‐existing disease (diabetes, hypertension, liver, kidney, thyroid or cardiovascular disease)	ADA criteria, at 24–28 weeks' gestation.	PCOS, normal BMI <25 and overweight/obese BMI ≥ 25	Not reported
		Cohort 2 *n* = 18,106; PCOS *n* = 877, controls *n* = 17,229	Cohort 2 February 2016–December 2017					
Sánchez‐García 2020, USA	Prospective observational	*n* = 164; GDM *n* = 29 (17.7%), non‐GDM *n* = 135 (82.3%)	November 2017–October 2019	Maternal age 18–35 years; in 1st trimester of pregnancy (<14 weeks according to last menstrual period)	Maternal age <18 years, taking any medications or had any illness that could impair insulin secretion/action (e.g., prediabetes, types 1 and 2 diabetes, PCOS Rotterdam criteria [53]); multifetal pregnancy; previous GDM or pre‐eclampsia	IADPSG criteria, at 24–28 weeks' gestation.	Triglycerides, BMI	BMI, parity, family history, diastolic blood pressure
Hashemi‐Nazari 2020, Iran	Retrospective cohort	*n* = 1010; analysed *n* = 1009; GDM *n* = 80, non‐GDM *n* = 929	2015–2016	Pregnant women referred to 10 health centres. A certain number of pregnant women	Not reported	ADA criteria, at 24–28 weeks' gestation.	BMI	Age, parity, family history of T2DM, abortion
Wani 2020, Saudi Arabia	Longitudinal prospective cohort	*n* = 498; GDM *n* = 123 (24.7%)	Not reported	Normal pregnant Saudi women; age 18–35 years; early pregnancy (<15 weeks' gestation); singleton pregnancy	Known previous multiple pregnancy; history of diabetes or chronic disease for example, renal or liver disease	IADPSG criteria, at 27 weeks' gestation	WC, fasting glucose, HDL‐C, TG, SBP, DBP, MetS	Age, BMI, parity
Berggren 2017, USA	Prospective observational	*n* = 300; analysed *n* = 250; GDM *n* = 72, non‐GDM *n* = 178	June 2012–June 2013	Maternal age ≥16 years; singleton pregnancy; gestational age 11–14 weeks; no known type 2 diabetes; planned care and delivery at study site; English proficiency	Known type 2 diabetes	Carpenter and Coustan criteria, at 22 0/7 to 33 6/7 weeks of gestation.	HbA1c, SHBG, BMI	HbA1c, SHBG, race, BMI, history of GDM
Grewal 2012, India	Prospective observational	Initial Cohort (12 weeks' gestation) *n* = 298; GDM *n* = 24	July 2006–January 2009	Non‐diabetic women; registered at antenatal clinic <12 weeks' gestation	History of overt diabetes; impaired fasting glucose or impaired glucose tolerance at initial prenatal visits; history of GDM or preeclampsia; taking medications known to affect BGL and insulin levels	Carpenter and Coustan criteria, at 24–28 weeks' gestation.	Early pregnancy plasma glucose, insulin, whole body insulin sensitivity, HOMA‐IR, QUICKI	Age and BMI
		Repeat Cohort (24 weeks' gestation) *n* = 215; GDM *n* = 16						
		Total Cohort *n* = 298; GDM *n* = 40						
Ogonowski 2007, Poland	Retrospective analysis	*n* = 2425; GDM *n* = 1414, non‐GDM control *n* = 1011	January 1999–December 2005	Pregnant women with abnormal OGTT, referred to the Outpatient Clinic for Diabetic Pregnant	Pre‐pregnancy diabetes	WHO criteria 1999, at 24–28 weeks' gestation.	Fasting plasma glucose	Not reported
Arbib 2019, Israel	Retrospective cohort	First trimester *n* = 142; GDM *n* = 42, non‐GDM *n* = 100	1 August 2007–31 December 2014	Healthy singleton foetus with no known chromosomal or anatomic malformation and known maternal and neonatal short‐term pregnancy outcome	Previous diagnosis of type 1 or 2 diabetes, HbA1C ≥ 6.5%, and/or fasting plasma glucose ≥126 mg/dl; women whose glucose levels had already been tested <24 weeks of pregnancy, or no GDM screening or testing	Carptenter and Coustan criteria, at 24–28 weeks' gestation	HbA1C	Not reported
Teede 2011, Australia	Retrospective cohort	Early pregnancy (12–15 weeks' gestation) *n* = 2880; GDM *n* = 250, non‐GDM *n* = 2630	2007–June 2008	All pregnant women (*n* = 4276) who delivered at Monash Medical Centre	Not reported	ADIPS criteria, at 26–28 weeks' gestation	BMI	Age, increasing BMI, ethnicity, first‐degree family history of diabetes, past history of GDM and/or history of poor obstetric outcome, ethnicity
Punnose 2020, India	Retrospective cohort	First trimester (13 6/7 weeks) *n* = 2275; GDM *n* = 578, non‐GDM *n* = 1697	January 2011–December 2016	Pregnant women with singleton pregnancies, HbA1c in 1st trimester	Twins, delivery outside the study hospital, HbA1c >6.5%, previous DM, Haemoglobinopathy	IADPSG criteria, at 24–28 weeks' gestation.	HbA1C	Age, BMI, previous GDM, family history of DM, multigravidity, Hb, MCV
Berggren 2015, USA	Prospective	Women in pre‐natal care or seeking a first trimester ultrasound *n* = 250; glucose intolerant *n* = 72, normoglycemic *n* = 178	June 2012–June 2013	Age ≥16 years; singleton pregnancies at 11 0/7 to 14 6/7 weeks; planned care and delivery at the study site; English language proficiency	No known history of type 2 diabetes mellitus; if GDM screening was performed at an earlier gestational age and GDM was then either diagnosed or treated based on that early screening, or if repeat GDM screening was not performed in the study‐specific gestational age window.	Carpenter and Coustan criteria, at 22–34 weeks' gestation.	HbA1C	SHBG, race, BMI, history of GDM.
Li 2016, China	Prospective	Women with PCOS in first pre‐natal visit (<15 weeks' gestation) *n* = 248; GDM *n* = 75, non‐GDM *n* = 173	2011–2013	18–45 years, diagnosis of PCOS before conception, singleton pregnancy	Pre‐existing chronic diseases including diabetes, hypertension, thyroid, kidney or cardiovascular disease, or multiple pregnancies	ADA criteria, at 24–28 weeks' gestation.	BMI, SBP, DBP, TC, TG, HDL‐C	FPG, non‐HDL‐c, SHBG
Riskin‐Mashiah 2010, Israel	Retrospective	*n* = 4876; GDM *n* = 135 (2.8%), non‐GDM *n* = 4741	June 2001–June 2006	Singleton pregnancy; 1st trimester BMI; 1st trimester fasting plasma glucose level	Pre‐gestational DM; fasting glucose level >105 mg/dl; delivery at <24 weeks' gestation	Carpenter and Coustan criteria, at 24–28 weeks' gestation.	BMI, fasting glucose	Fasting glucose level, BMI, maternal age, parity
Raja 2012, UK	Retrospective	*n* = 27,668; BMI<30 kg/m^2^; *n* = 20,735, BMI>30 kg/m^2^; *n* = 3897	January 2002–December 2007	Delivering at Northwick Park Hospital, Harrow; delivery date between 1 January 2002 and 31 December 2007	Lack of data on weight/height	Not reported (hospital database)	BMI	Maternal age, ethnicity, parity, cigarette smoking
Gabbay‐Benziv 2015, USA	Prospective cohort	*n* = 927; GDM *n* = 63 (6.8%), non‐GDM *n* = 861	2007–2010	Baltimore metropolitan area; singleton intrauterine pregnancy between 11 and 14 weeks' gestation; prenatal care and subsequent GDM screening at study centre	Strong evidence for pre‐GDM, and missing outcomes	Carpenter and Coustan criteria, at 24–28 weeks' gestation.	BMI, BP	Maternal age, ethnicity, prior GDM, first trimester BMI, SBP
Basraon 2016, USA	Prospective cohort	*n* = 2300; GDM *n* = 80 (3.5%), non‐GDM *n* = 2220	2003–2008	Singleton pregnancy; 9–16 weeks' gestation; nulliparous women; no history of pre‐gestational hypertension, proteinuria, diabetes or other medical problems; substance abuse; foetal abnormalities; uterine bleeding; in‐vitro fertilisation	No data of WHR and BMI	GDM diagnosis at 26 weeks' gestation as per the guideline of each centre	BMI, WHR, insulin resistance	Maternal age, education, ethnicity, weeks of gestation at enrolment, alcohol, smoking status
Alptehkin 2016, Turkey	Prospective observational	*n* = 227; GDM *n* = 20 (8.8%), non‐GDM *n* = 207	December 2014–May 2015	Singleton pregnancy; 7–14 weeks' gestation	Previous type:1 or 2 diabetes, with FPG >95 mg/dl, multiple pregnancies, untreated endocrine disturbances, chronic hypertension, preeclampsia, or medication that affected fasting glucose or insulin levels	Carpenter and Coustan criteria, at 24–28 weeks' gestation.	HOMA‐IR, BMI	BMI, WHR, parity, weight gain during pregnancy, HOMA‐IR
Li 2019, China	Retrospective	*n* = 2112; GDM *n* = 224 (10.6%), non‐GDM *n* = 1888	January 2016–June 2017	First prenatal visit during 9–13 + 6 weeks' gestation; regular prenatal services; delivered in third affiliated hospital of Sun Yat‐Sen University, Guangzhou, China	Diagnosed pre‐gestational diabetes	IADPSG criteria, at 24–28 weeks' gestation.	FPG	Pre‐pregnancy BMI, first‐trimester FPG, maternal age, parity
Wolfe 1991, USA	Prospective	*n* = 6270	30 month period, year not reported	Consecutively delivered of infants at Hutzel Hospital; antepartum and intrapartum records were available	Not reported	Not reported	BMI, maternal weight	Not reported
Nanda 2011, UK	Prospective, case‐control	*n* = 11,464; GDM *n* = 297 (2.6%), non‐GDM *n* = 11,167	March 2006–August 2009	Women who attended first antenatal visit 11–13 weeks' gestation; singleton pregnancy; delivered phenotypically normal neonate ≥30 weeks' gestation	Pre‐pregnancy type 1 or 2 diabetes; termination, miscarriage or delivery <30 weeks'	WHO criteria 2006, at 24–28 weeks' gestation.	BMI	Maternal age, race, family history of diabetes, parity, cigarette smoking, conception
Kumru 2016, Turkey	Prospective cohort	*n* = 333; GDM *n* = 38, non‐GDM *n* = 295	January 2011–January 2013	Provided blood samples at 6–13 ± 6 weeks' gestation; completed prenatal care; delivered a live, term infant at institution	Multiple pregnancies; obesity (BMI > 30 kg/m^2^); history of hypertension; type 1/2 diabetes or glucose intolerance pre‐pregnancy; GDM; preeclampsia; intrauterine 2nd or 3rd trimester pregnancy loss; first‐ or second‐degree relative with diabetes; 1st^,^ 2nd^,^ 3rd trimester losses during follow up; foetal anomaly; did not complete pre‐natal care or deliver at hospital	Carpenter and Coustan criteria, at 24–28 weeks' gestation.	BMIs, MAPs, FBG, insulin, HbA1c, HOMA, TC, LDL‐C, and TG	Maternal age, 1st trimester BMI, MAP
Hancerliogullari 2020, Turkey	Prospective cohort	*n* = 525; GDM *n* = 49 (9%), non‐GDM *n* = 476 (91%)	August 2018–November 2018	Low‐risk pregnant women at 11–14 weeks' gestation.	Maternal age <18 years or >45 years, multiple pregnancies, women with known hypertension, kidney, liver, thyroid gland and other endocrine diseases, those who were diagnosed with pre‐diabetes	Carpenter and Coustan criteria, at 24–28 weeks' gestation.	WC, BMI	Not reported
Ozgu‐Erdinc 2019, Turkey	Retrospective cohort study	*n* = 439; GDM *n* = 49 (11.2%)	January 2011–January 2012	Patients who had received antenatal care during 1st trimester	Multiple gestations, medications that affect insulin and glucose levels, hypertension or concomitant systemic disease, pre‐gestational known diabetes (type 1–2) or glucose intolerance and FPG levels ≥126 mg/dl. Four women were excluded as lost to follow‐up	ACOG criteria, at 24–28 weeks' gestation.	FPG	FPG, insulin ratio, HOMA‐IR, HOMA‐b indices, QUICKI
Gao 2020, China	Prospective cohort	Training dataset *n* = 12,887; GDM *n* = 979 (7.6%). Test dataset *n* = 6444; GDM *n* = 506 (7.9%)	October 2010–August 2012	19,331 pregnant women registered for antenatal care and two‐step GDM screening. Dataset was randomly divided into two using a computer‐generated random number: The training dataset and the test dataset, with the ratio of sample size of 2:1. The training dataset was used to develop the risk score and the test dataset was used to validate.	History of type 1 or 2 diabetes before pregnancy, 936 who registered and attended their first antenatal care in more than the 15th gestational week, 1163 women who did not undergo GCT, and 851 women who had a positive GCT but did not undergo OGTT.	Changed from WHO 1999 criteria to IADPSG criteria in 2010, at 24–28 weeks of gestation.	BMI, SBP, DBP, weight, WC	Not reported
Liu 2020, China	Prospective	Singleton pregnancy *n* = 352; GDM *n* = 66 (18.8%), non‐GDM *n* = 286	October 2018–December 2018	Singleton pregnancy, followed up prospectively from the first prenatal visit until delivery.	Not a singleton pregnancy; not Han ethnicity; fasting glucose ≥6.1 mmol/L and/or HbA1c >6.5% or diagnosed as diabetes before pregnancy; history of autoimmune disease, or currently use corticosteroids; hyperthyroidism or hypothyroidism; miscarried or induced labour before OGTT at 24–28 weeks; history of liver or renal insufficiency or CRP >10 mg/L; suspected familial hypertriglyceridemia; incomplete records of lipid profiles and/or FPG concentration.	IADPSG/WHO criteria, at 24–28 weeks of gestation.	BMI, TG, HDL‐C, TC	Age, education, physical activity, BMI (at enrolment), parity, family history of diabetes, history of PCOS, CRP, labour method, foetal sex, gestation age and weight gain
Meek 2021, UK	Retrospective	Older Cambridge University Hospital	2004–2008	*n* = 17,736 consecutive women with singleton pregnancies, with random plasma glucose at booking	Not reported	UK National Institute for Health and Care Excellence (NICE; 0 min = 5.6 mmol/l; 120 min = 7.8 mmol/l) and the IADPSG, adopted by the WHO; 0 min = 5.1; 60 min = 10.0; 120 min = 8.5 mmol/l, at 28 weeks' gestation.	Glucose	Not reported
		NHS Foundation Trust cohort, *n* = 17,736; GDM cases not specified						
Guo 2020, China	Retrospective cohort was used to develop a prediction model which was assessed on a prospective cohort study	Retrospective *n* = 3956; GDM *n* = 662, non‐GDM *n* = 3294	January 2015–December 2015	Eligible subjects who underwent 1st‐trimester screening at the International Peace Maternity and Child Care Health Hospital were recruited at 9–13 weeks' gestation	Pre‐existing diabetes (FPG ≥7 mmol/L or HbA1c ≥ 6.5% during the first antenatal care or self‐reported previous diabetes), multifetal pregnancies, missing data	ADA criteria, at 24–28 weeks' gestation.	FPG, HbA1c	Advanced age, high pre‐pregnancy BMI, diabetes in first degree relatives
Wang 2016, China	Retrospective cohort	*n* = 15,194; included *n* = 5265; GDM *n* = 1062, non‐GDM *n* = 4203	20 June 2013–30 November 2013	Singleton pregnancies delivered between 20 June 2013, and 30 November 2013;	Pre‐existing diabetes mellitus (*n* = 209), multiple births (*n* = 253), missing data on early pregnancy lipid and fasting glucose concentrations (*n* = 9467).	China recommendations: When any one value met or exceeded a 0 h glucose level of 5.1 mM, a 1 h glucose level of 10.0 mM, and a 2 h glucose level of 8.5 mM after a diagnostic 75 g OGTT between 24 and 28 weeks' gestation.	Fasting glucose, TC, TG	Age, family history of DM
Al‐Shafei 2021, Sudan	Nested case‐control	GDM: 60, non‐GDM:60	January–November 2017	Singleton pregnancies who attended the prenatal care clinic of the hospital during early pregnancy (≤14 weeks' gestation).	Pregnant women with any chronic disease (e.g., diabetes or history of GDM, hypertension, renal disease, liver disease, or thyroid disease) and women who were on medication were excluded	IADPSG, at 24–28 weeks' gestation	FBG, BMI	Not reported
Zhang 2020, China	Prospective cohort	GDM: 274, non‐GDM:1111	December 2017–March 2019	Recruited at 7–12 weeks' gestation	Not reported	IADPSG criteria, at 24–28 weeks' gestation.	FBG, HbA1c, HDL‐C, SBP, DBP, TG, BMI	Age, BMI, and parity
Tenenbaum‐Gavish 2020, Israel	Prospective cohort	GDM:20; non‐GDM:185	October 2014 and March 2016	Singleton viable gestation when undergoing combined first trimester screening for aneuploidy. Patients with placentation support hormonal treatment for in vitro fertilisation were only included after discontinuing treatment.	Foetal aneuploidies or major foetal anomalies, increased nuchal translucency thickness >3.5 mm or treatment with aspirin prior to enrolment; termination, miscarriage, or foetal death before 24 weeks' gestation, pre‐eclampsia, birthweight <5th percentile for gestational age, delivered <37 weeks' gestation	Carpenter and Costan criteria, at 24–28 weeks' gestation.	BMI, SBP, DBP	Not reported
Leng 2015, China	Prospective cohort	GDM: 1378; non‐GDM: 16,430; within 12 weeks of gestation	October 2010–August 2012	Not reported	Women who did not have GCT at 24–28 weeks' gestation	1999 WHO criteria, at 24–28 weeks' gestation.	BMI, SBP, DBP	Age, BMI, and parity, Han nationality, SBP, family history of diabetes in first degree family, education, weight gain from pre‐pregnancy to GCT, smoking and drinking habits
Schneider 2021, Australia and New Zealand	Prospective analysis	GDM: 184; non‐GDM: 974	March 2015–December 2017	Not reported	Not reported	WHO 2013 criteria, classification, at 24–28 weeks' gestation.	MetS	Maternal BMI, age, ethnicity, SEI, pre‐pregnancy fast food intake, pre‐pregnancy fruit intake, smoking status

*Note*: Criteria used for GDM diagnoses:

WHO 1999: Fasting glucose ≥7.0 mmol/L (126 mg/dl); ≥7.8 mmol/L (140.4 mg/dl) for 2‐h plasma glucose.

IADPSG, 2010/WHO 2013: Fasting plasma glucose = 5.1–6.9 mmol/L (92–125 mg/dl); 75 g oral glucose load: 1‐h ≥10.0 mmol/L (180 mg/dl), 2‐h 8.5–11.0 mmol/L (153–199 mg/dl).

Carpenter‐Coustan/ADA: 100 g oral glucose load: Fasting, 95 mg/dl (5.3 mmol/L), 1‐h, 180 mg/dl (10.0 mmol/L), 2 h, 155 mg/dl (8.6 mmol/L), and 3 h, 140 mg/dl (7.8 mmol/L).

Australasian Diabetes in Pregnancy: Fasting blood glucose level (BGL) ≥5.5 mmol/L (100 mg/dl) and/or 1‐h BGL ≥10.5 mmol/L (190 mg/dl) and/or 2‐h BGL ≥8.0 mmol/L (144 mg/dl); or a screening 50 g glucose challenge test (GCT) and if positive (1‐h BGL ≥7.8 mmol/L [140 mg/dl]), a subsequent OGTT.

American College of Obstetrics and Gynaecologists: Fasting plasma glucose: ≥5.3 mmol/L; 100 g OGTT: 1‐h plasma glucose ≥10.0 mmol/L, and 2‐h plasma glucose ≥8.6 mmol/L.

National Diabetes Group: Fasting, 1‐h, 2‐h, and 3‐h plasma glucose levels of 105 mg/dl (5.8 mmol/l), 190 mg/dl (10.5 mmol/L), 165 mg/dl (9.2 mmol/L), and 145 mg/dl (8.0 mmol/L).

In meta‐analysis, the minimum set of confounding variables included were: maternal age for overweight and obesity analyses; maternal age and BMI for fasting plasma glucose (FPG), TG, HbA1c, HDL‐C, and MetS analyses; and maternal age, BMI, ethnicity, and family history of diabetes, for systolic blood pressure analyses.

### 3.2 Quality assessment

Supporting Information (Table S1) presents the risk of bias using the QUIPS tool for each of the 78 included articles. The overall risk of bias was judged as high for 31 studies (39.7%), moderate for 25 studies (32.0%) and low for 22 studies (28.2%). For individual criteria, 33 studies (42.3%) and 31 studies (39.7%), respectively, were graded as high risk for ‘Adjustment for other prognostic factors’ and ‘Statistical analysis and reporting’. One third of the studies reported a diagnosis of GDM using the 2010 International Association of the Diabetes and Pregnancy Study Groups criteria,^50^ which was later adopted by the World Health Organization in 2013^51^; around a fifth of studies used the Carpenter and Coustan criteria,^52^ and around a quarter of included studies did not report on the diagnosis criteria, or used criteria from within their own institution.

### 3.3 Narrative review and meta‐analysis

Supporting Information (Figures S1–S7) illustrates the narrative results reporting on mean differences in each metabolic factor between women with and without GDM. Figures 2, 3, 4, 5, 6, 7, 8, 9, 10 present the OR (95% CI) of maternal prognostic metabolic factors in early pregnancy and the likelihood for GDM. The results of the individual factors are summarised below.

#### 3.3.1 Waist circumference (WC)

Six cohort studies assessed WC with sample sizes ranging from 247 to 19,186.^53^, ^54^, ^55^, ^56^, ^57^, ^58^ Overall, women who developed GDM had a larger WC measured in early pregnancy, with a mean difference of 6.20 cm compared to women without GDM (*p* < 0.0001; Supplementary Figure S1).^56^, ^57^, ^58^ Studies were not pooled in the meta‐analysis as they did not report on OR or RR.

#### 3.3.2 Body mass index (BMI)

Body mass index was derived from recorded medical history data, self‐report, or from a measurement at the first antenatal visit. There were 44 cohort and six nested case‐control studies, with sample sizes ranging from 106 to 132,899 participants.^49^, ^53^, ^54^, ^55^, ^56^, ^57^, ^58^, ^59^, ^60^, ^61^, ^62^, ^63^, ^64^, ^65^, ^66^, ^67^, ^68^, ^69^, ^70^, ^71^, ^72^, ^73^, ^74^, ^75^, ^76^, ^77^, ^78^, ^79^, ^80^, ^81^, ^82^, ^83^, ^84^, ^85^, ^86^, ^87^, ^88^, ^89^, ^90^, ^91^, ^92^, ^93^, ^94^, ^95^, ^96^, ^97^, ^98^, ^99^, ^100^, ^101^ Overall, the mean BMI was 2.28 kg/m^2^ higher in women with GDM compared to women without GDM (*p* < 0.00001; Supplementary Figure S2).^53^, ^56^, ^57^, ^58^, ^59^, ^60^, ^61^, ^62^, ^63^, ^64^, ^65^, ^66^, ^98^, ^99^, ^100^, ^101^

**FIGURE 2:**
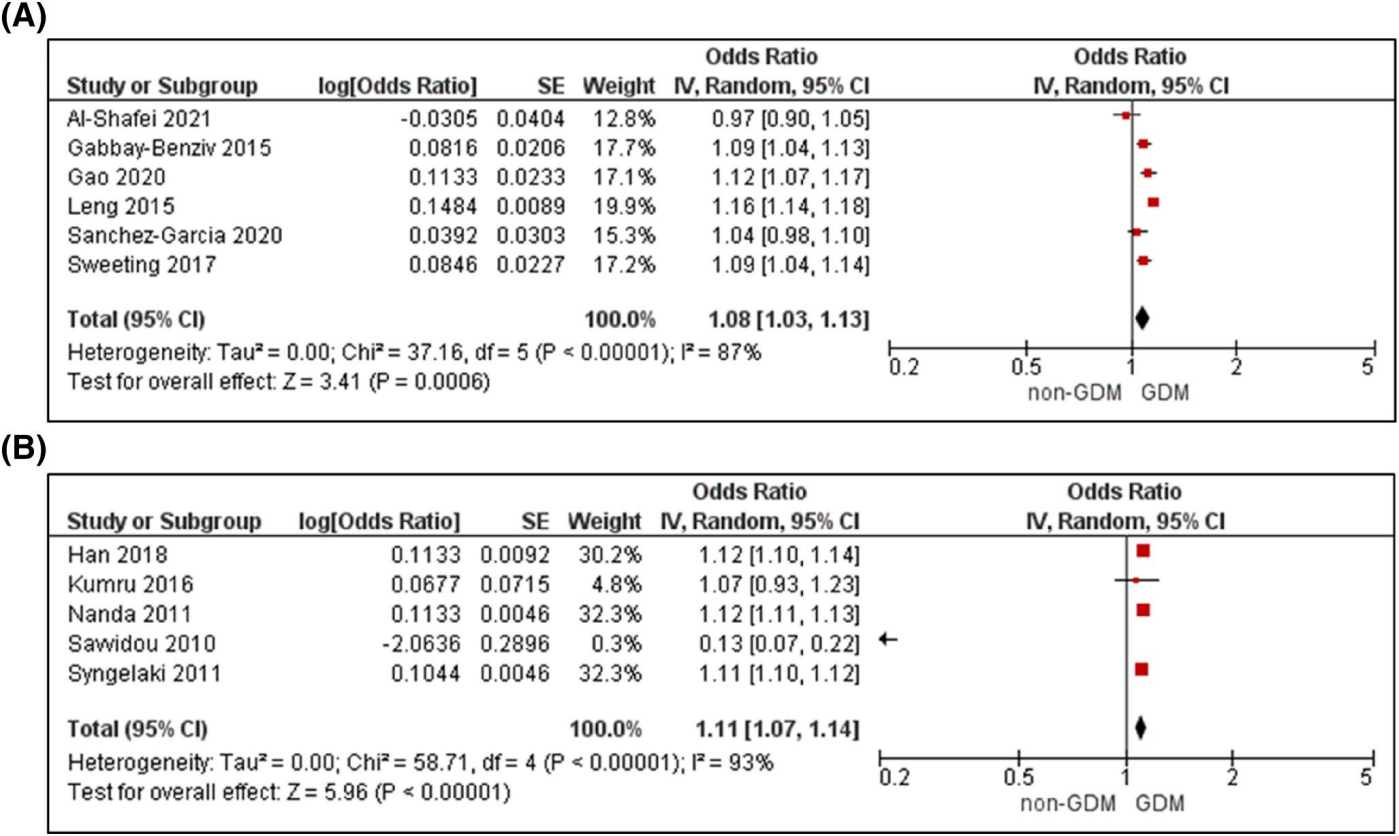
Meta‐analysis of early pregnancy body mass index (BMI) and odds of gestational diabetes. Values are odds ratios (OR) with 95% confidence intervals (CIs) for (A) unadjusted and (B) adjusted for maternal age, analyses. For overall effect, *p*‐value <0.05 was considered significant

Seven cohort and five case‐control studies with continuous BMI data were included in meta‐analyses.^55^, ^58^, ^61^, ^66^, ^67^, ^68^, ^69^, ^70^, ^71^, ^72^, ^98^, ^99^ For every unit increase in BMI, there was a slight increase in odds for GDM in unadjusted analysis (OR 1.08, 95% CI 1.03–1.13, *k* = 6; Figure 2A) and analyses adjusted for age, as a key criterion, and up to another 10 confounders (aOR 1.11, 95% CI 1.07–1.14, *k* = 5; Figure 2B). There was clear heterogeneity across the studies (*I* ^2^ ≥ 87%, *P*_het_ < 0.00001 for both).

**FIGURE 3:**
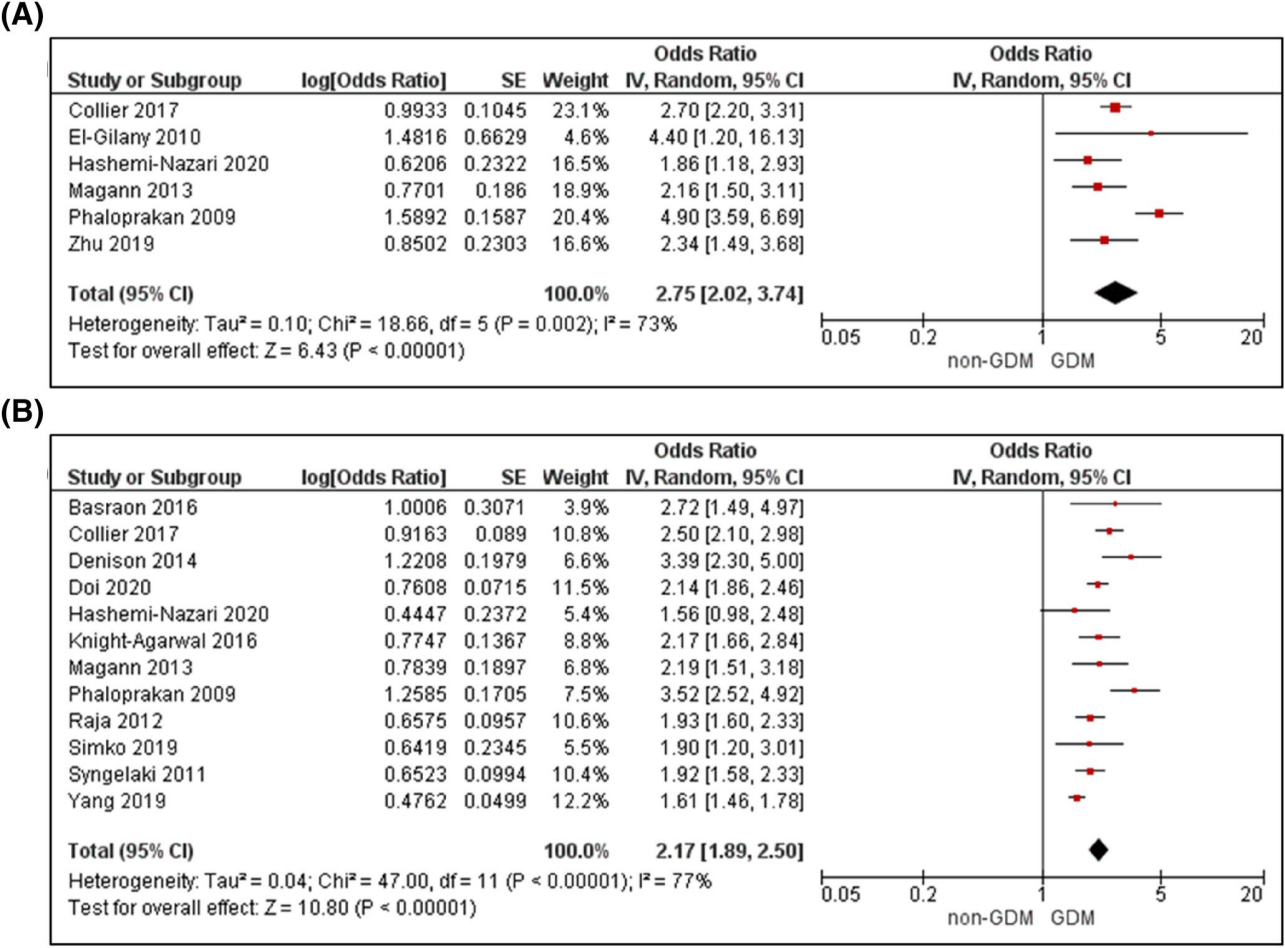
Meta‐analysis of early pregnancy overweight and odds of gestational diabetes. Values are odds ratios (OR) with 95% confidence intervals (CIs) for (A) unadjusted and (B) adjusted for maternal age, analyses. For overall effect, *p*‐value <0.05 was considered significant

**FIGURE 4:**
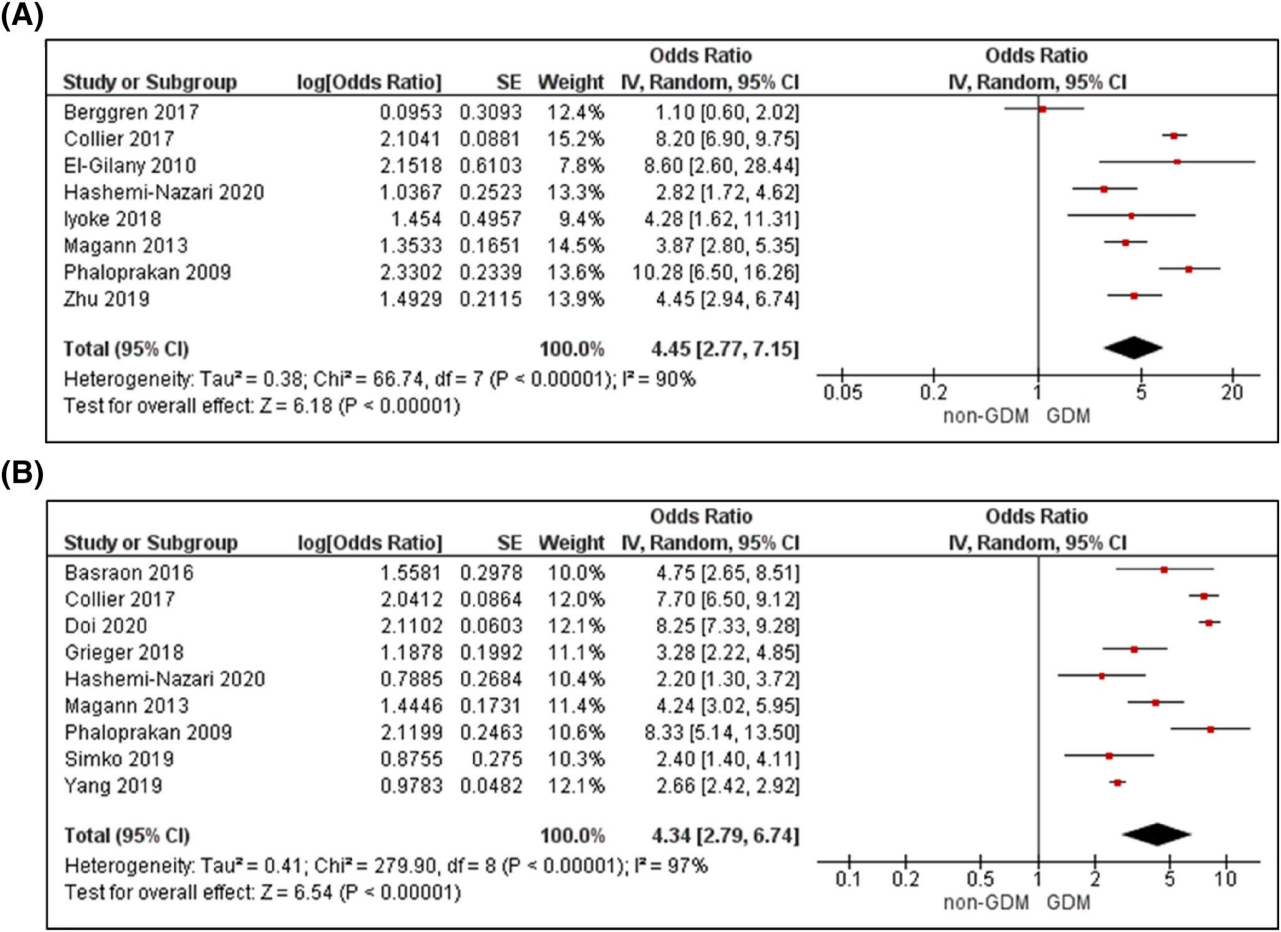
Meta‐analysis of early pregnancy obesity and odds of gestational diabetes. Values are odds ratios (OR) with 95% confidence intervals (CIs) for (A) unadjusted analysis and (B) adjusted for maternal age. For overall effect, *p*‐value <0.05 was considered significant

Fourteen studies reported on an overweight BMI (>25–<30 kg/m^2^),^49^, ^54^, ^59^, ^68^, ^78^, ^79^, ^80^, ^81^, ^83^, ^84^, ^86^, ^87^, ^89^, ^91^ demonstrating a 2‐3 fold increased odds for GDM in unadjusted analyses (OR 2.75; 95% CI 2.02–3.74, *k* = 6; Figure 3A) and analyses adjusted for age, and up to four other confounders (OR 2.17; 95% CI 1.89–2.50, *k* = 12; Figure 3B). There was high statistical heterogeneity (*I* ^2^ ≥ 73%, *P*_het_ ≤ 0.002 for both). For the 13 studies including women with obesity (BMI > 30 kg/m^2^),^24^, ^49^, ^54^, ^59^, ^78^, ^81^, ^83^, ^86^, ^87^, ^89^, ^91^, ^93^, ^94^ an obese BMI was associated with a 4‐fold increased odds of GDM (unadjusted OR 4.45; 95% CI 2.77–7.15, *k* = 8, Figure 4A; adjusted OR 4.34; 95% CI 2.79–6.74, *k* = 9, Figure 4B), with high statistical heterogeneity (*I* ^2^ ≥ 90%, *P*_het_ < 0.00001 for both).

#### 3.3.3 Blood pressure

Ten cohort and two case‐control studies provided data on systolic (SBP), diastolic (DBP), and mean arterial blood pressure with sample sizes ranging from 205 to 17,808 participants.^24^, ^53^, ^58^, ^64^, ^66^, ^69^, ^85^, ^99^, ^100^, ^101^, ^102^, ^103^ Women with GDM had 3.15 mmHg higher mean SBP (*p* < 00001; Supplementary File 2)^53^, ^58^, ^64^, ^66^, ^99^, ^100^, ^101^, ^102^ and 1.78 mmHg higher mean DBP (*p* < 00001; Supplementary Figure S3)^53^, ^58^, ^64^, ^66^, ^99^, ^100^, ^101^, ^102^ compared to women without GDM.

**FIGURE 5:**
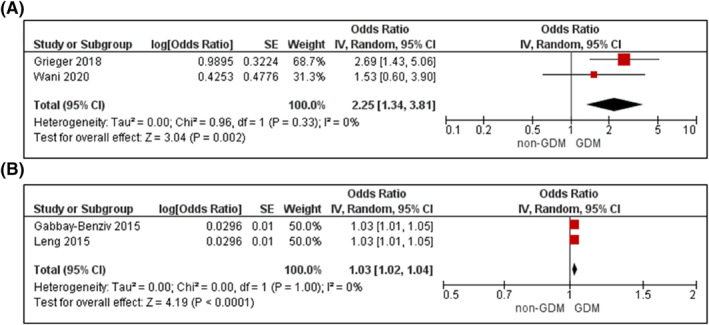
Meta‐analysis of blood pressure during early pregnancy and odds of gestational diabetes. Values are odds ratios (OR) with 95% confidence intervals (CIs) for (A) raised blood pressure (systolic blood pressure >130 mm Hg or diastolic blood pressure >85 mm Hg) and (B) systolic blood pressure; adjusted for maternal age, BMI, family history, and ethnicity. For overall effect, *p*‐value <0.05 was considered significant

Raised blood pressure (SBP > 130 mmHg or DBP > 85 mmHg)^24^, ^53^ or raised SBP^76^, ^106^ was associated with increased odds for GDM (OR 2.25 95% CI 1.34–3.81, *k* = 2, adjusted for age, BMI, and up to another seven confounders, Figure 5A; aOR 1.03, 95% CI 1.02–1.04, *k* = 2, adjusted for age, BMI, family history of diabetes, plus eight confounders, Figure 5B).^69^, ^99^ There was no statistical heterogeneity between studies for either analysis (both *I* ^*2*^ = 0%, *P*_het_ > 0.1).

#### 3.3.4 Fasting plasma glucose

**FIGURE 6:**
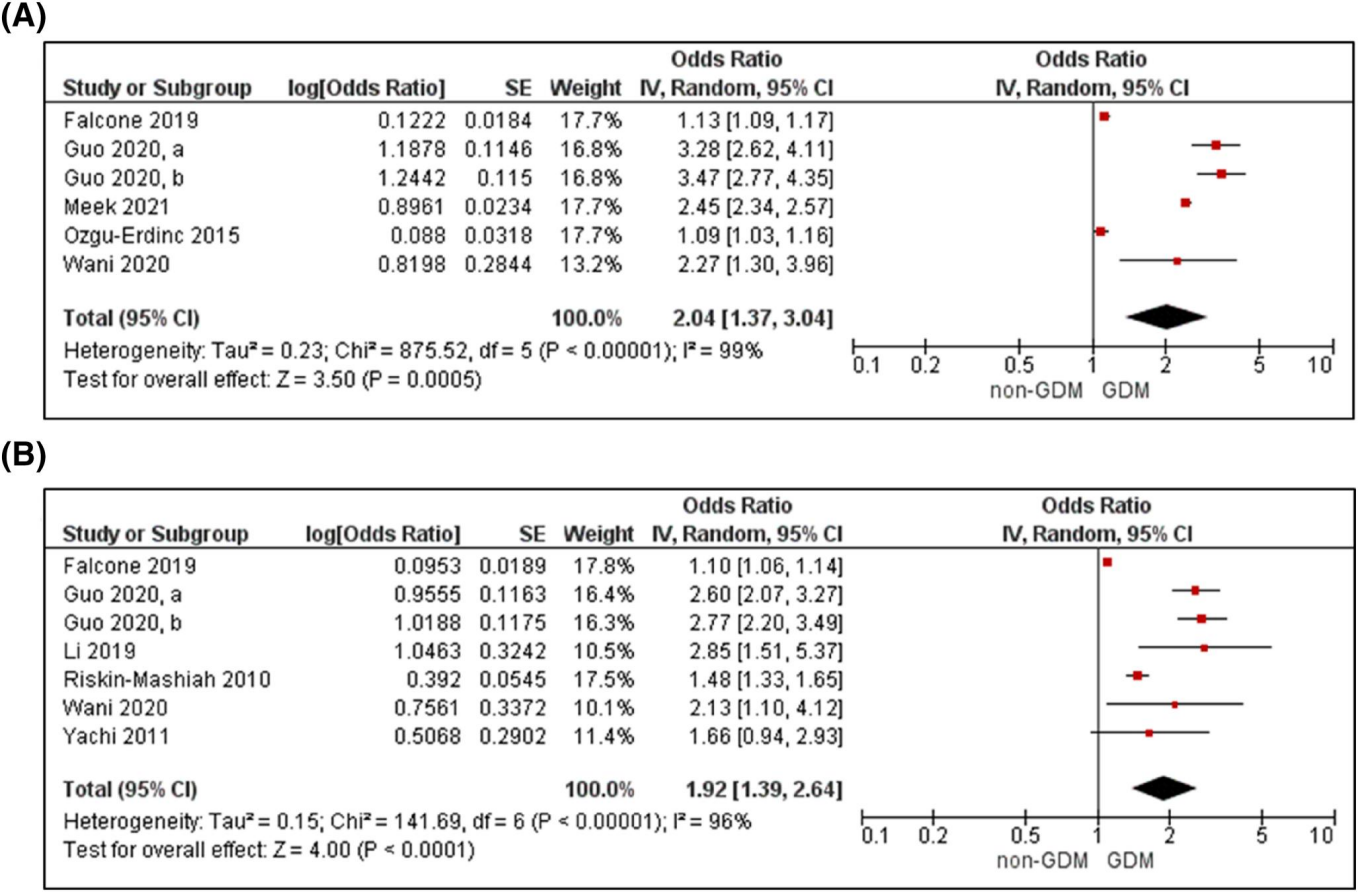
Meta‐analysis of early pregnancy fasting plasma glucose and odds of gestational diabetes. Values are odds ratios (OR) with 95% confidence intervals (CIs) for (A) unadjusted and (B) adjusted for maternal age and BMI, analyses. For overall effect, *p*‐value <0.05 was considered significant

Seventeen cohort, three nested case‐control and two retrospective analyses provided data on FPG.^24^, ^48^, ^53^, ^60^, ^61^, ^62^, ^65^, ^72^, ^73^, ^85^, ^97^, ^98^, ^101^, ^102^, ^104^, ^105^, ^106^, ^107^, ^108^, ^109^, ^110^, ^111^ Sample sizes ranged from 106 to 17,736 participants. Overall, women with GDM had a mean 0.41 mmol/L higher FPG during early pregnancy compared to women without GDM (*p* < 00,001; Supplementary Figure S4).^48^, ^53^, ^60^, ^61^, ^65^, ^101^, ^102^, ^106^, ^107^, ^111^ Nine studies comprising eight unique samples were included in the meta‐analysis.^53^, ^60^, ^62^, ^72^, ^73^, ^97^, ^104^, ^105^ Increased FPG in early pregnancy was associated with a higher odds for GDM (unadjusted OR 2.04; 95%CI 1.37–3.04, *k* = 6, Figure 6A; adjusted for age, BMI, and up to seven other confounders, OR 1.92; 95% CI 1.39–2.64, *k* = 7, Figure 6B).

#### 3.3.5 Glycosylated haemoglobin (HbA1c)

**FIGURE 7:**
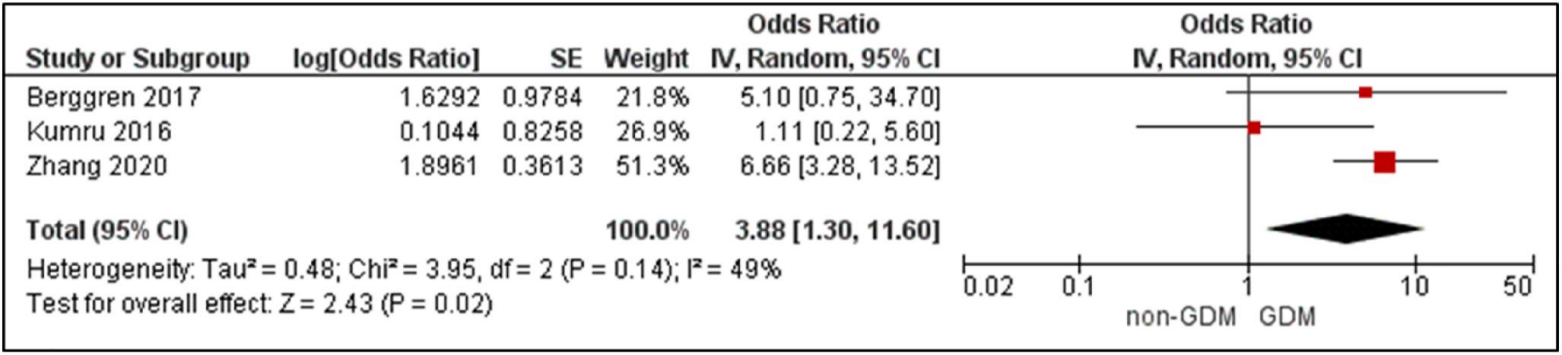
Meta‐analysis of early pregnancy glycosylated haemoglobin and odds of gestational diabetes. Values are odds ratios (OR) with 95% confidence intervals (CIs), adjusted for maternal body mass index (BMI). For overall effect, *p*‐value <0.05 was considered significant

Eight cohort, one case‐control, and one randomised controlled trial measured HbA1c in blood samples collected at the first antennal visit.^60^, ^61^, ^93^, ^101^, ^106^, ^112^, ^113^, ^114^, ^115^, ^116^ Sample sizes ranged from 142 to 2275 participants. Women who developed GDM had 0.20% higher mean HbA1c in early pregnancy compared to women without GDM (*p* < 0.0001; Supplementary Figure S5).^60^, ^61^, ^93^, ^101^, ^106^, ^112^, ^113^, ^114^ Three studies were used in the meta‐analysis,^61^, ^93^, ^101^ demonstrating an association between HbA1c and GDM (adjusted OR 3.88; 95% CI 1.30–11.60, *k* = 3; Figure 7, *I* ^2^ = 49%, *P*_het_ = 0.14).

#### 3.3.6 Triglycerides

Thirteen cohort and three nested case‐control studies measured fasting TG in early pregnancy.^24^, ^48^, ^53^, ^61^, ^63^, ^64^, ^65^, ^66^, ^85^, ^101^, ^107^, ^117^, ^118^, ^119^, ^120^, ^121^ Sample sizes ranged from 107 to 15,194 participants. Women who developed GDM had higher TG measured in early pregnancy compared to women who did not develop GDM (mean difference 0.24 mmol/L, *p* < 0.00001; Supplementary Figure S6).^48^, ^53^, ^61^, ^64^, ^65^, ^101^, ^107^, ^117^

**FIGURE 8:**
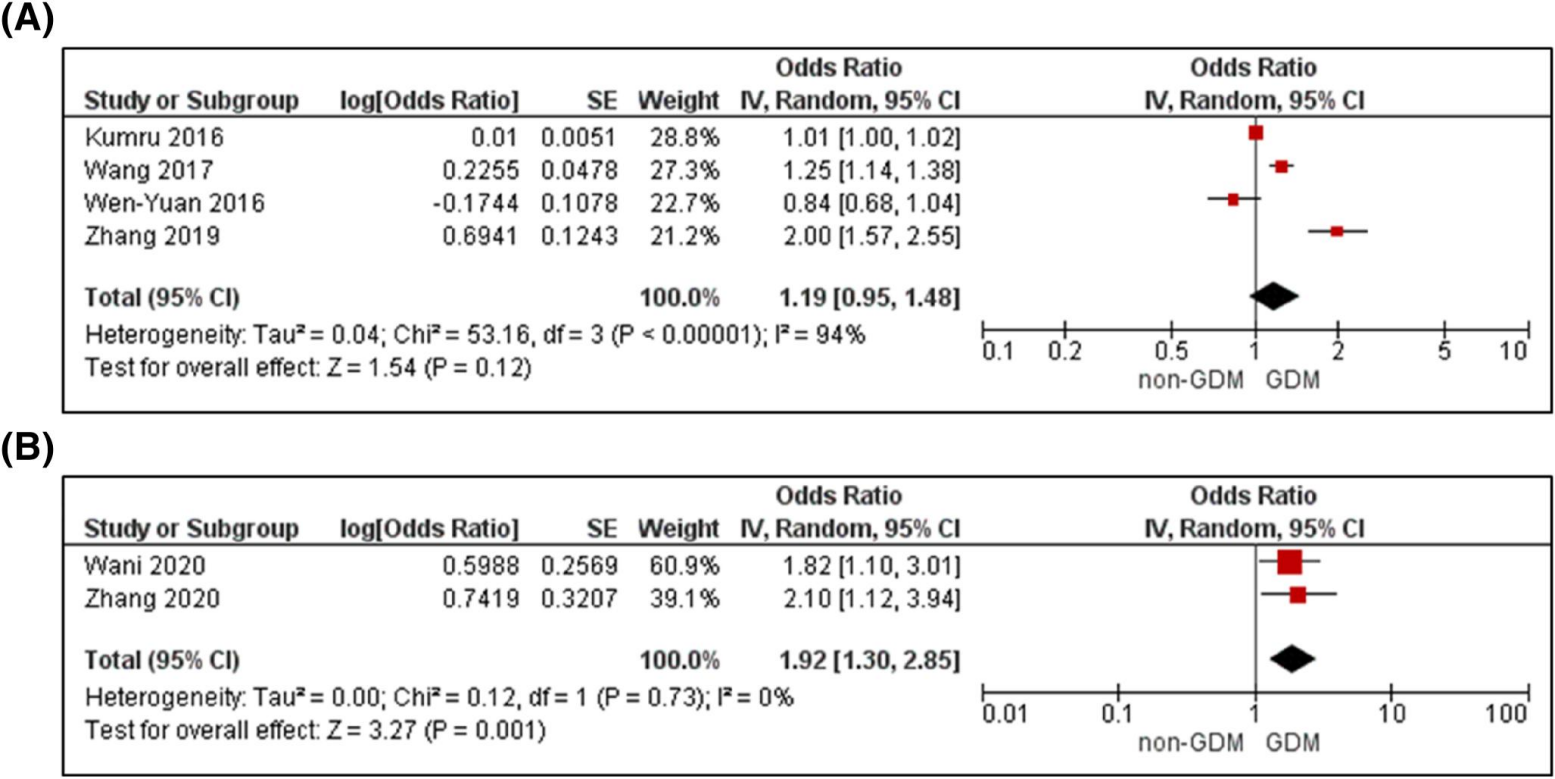
Meta‐analysis of early pregnancy triglycerides (TG) and odds of gestational diabetes. Values are odds ratios (OR) with 95% confidence intervals (CIs) (A) per one unit increase in TG and (B) TG > 1.7 mmol/l, adjusted for maternal age and body mass index (BMI). For overall effect, *p*‐value <0.05 was considered significant

Six studies were included in the meta‐analysis.^53^, ^61^, ^101^, ^117^, ^118^, ^119^ Increasing TG was associated with 1.19‐fold increased likelihood for GDM in analyses adjusted for age, BMI and up to another seven confounders (95% CI 0.95–1.48, Figure 8A) with high statistical heterogeneity between studies (*I* ^*2*^ = 94%, *P*_het_ < 0.00001, *k* = 4). Triglycerides >1.7 mmol/L was also associated with higher likelihood of GDM (adjusted OR 1.92; 95% CI 1.30–2.85, *I* ^*2*^ = 0%, *P*_het_ = 0.73, *k* = 2; Figure 8B).

#### 3.3.7 High‐density lipoprotein cholesterol (HDL‐C)

**FIGURE 9:**
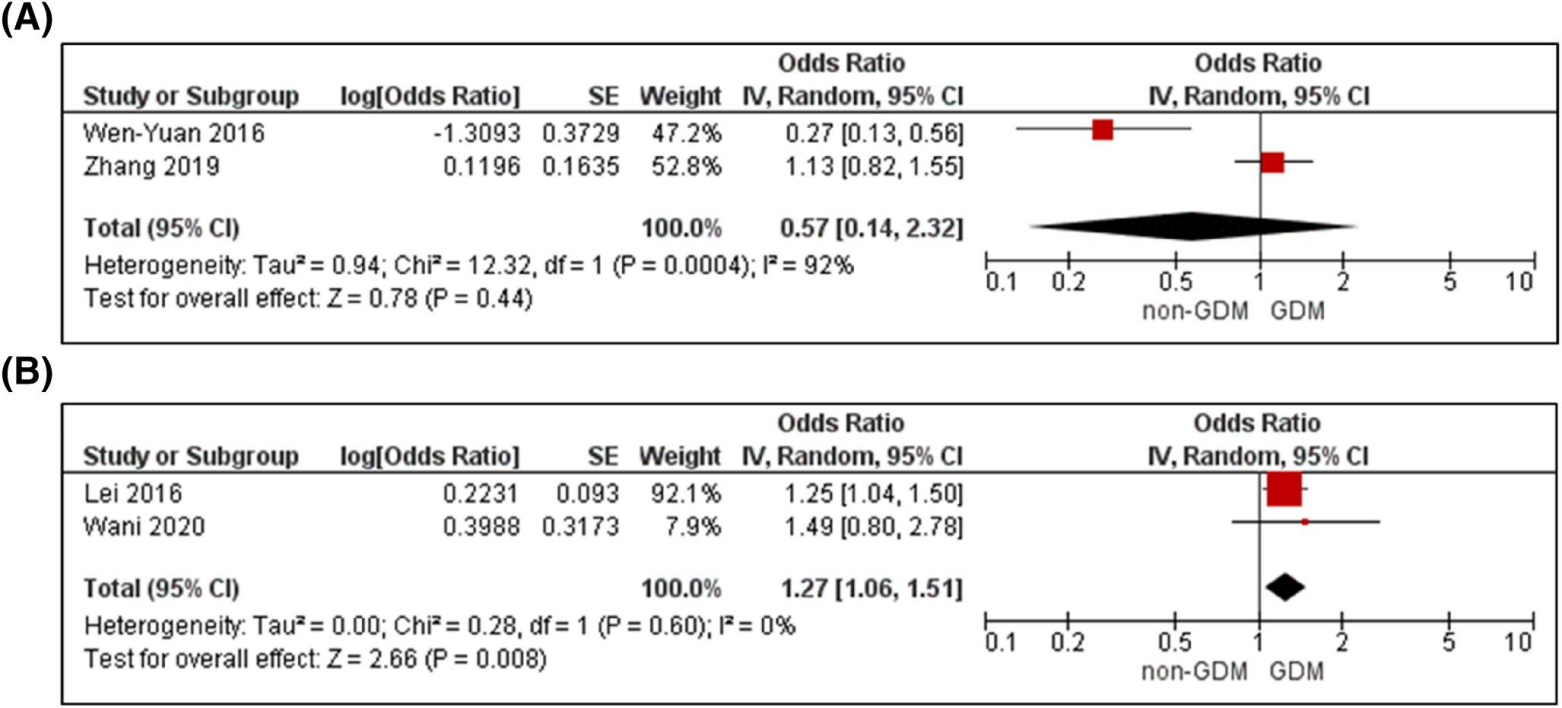
Meta‐analysis of early pregnancy high‐density lipoprotein cholesterol (HDL‐C) and odds of gestational diabetes. (A), Values are odds ratios (OR) with 95% confidence intervals (CIs) for one unit increase in HDL‐C adjusted for maternal age and body mass index (BMI). (B), Odds ratio with 95% CI for low HDL‐C (<1.3 mmol/l) adjusted for maternal age. For overall effect, *p*‐value <0.05 was considered significant

Eight cohort and 2 nested case‐control studies reported on fasting HDL‐C in early pregnancy.^53^, ^61^, ^63^, ^66^, ^85^, ^101^, ^117^, ^118^, ^119^, ^120^ Sample sizes ranged from 333 to 5218. Women with GDM had slightly lower mean HDL‐C compared to women without GDM (Supplementary File 2).^53^, ^61^, ^63^, ^66^, ^101^, ^117^ On meta‐analysis,^53^, ^85^, ^118^, ^119^ there was insufficient evidence to confirm an association between HDL‐C and GDM (aOR 0.57, 95% CI 0.14–2.32, *I* ^*2*^ = 92%, *P*_het_ = 0.0004, *k* = 2; Figure 9A), but an HDL‐C of <1.3 mmol/L was associated with higher odds of GDM (aOR 1.27; 95% CI 1.06–1.51, *I* ^*2*^ = 0%, *P*_het_ = 0.60, *k* = 2; Figure 9B).

#### 3.3.8 Metabolic syndrome

**FIGURE 10:**
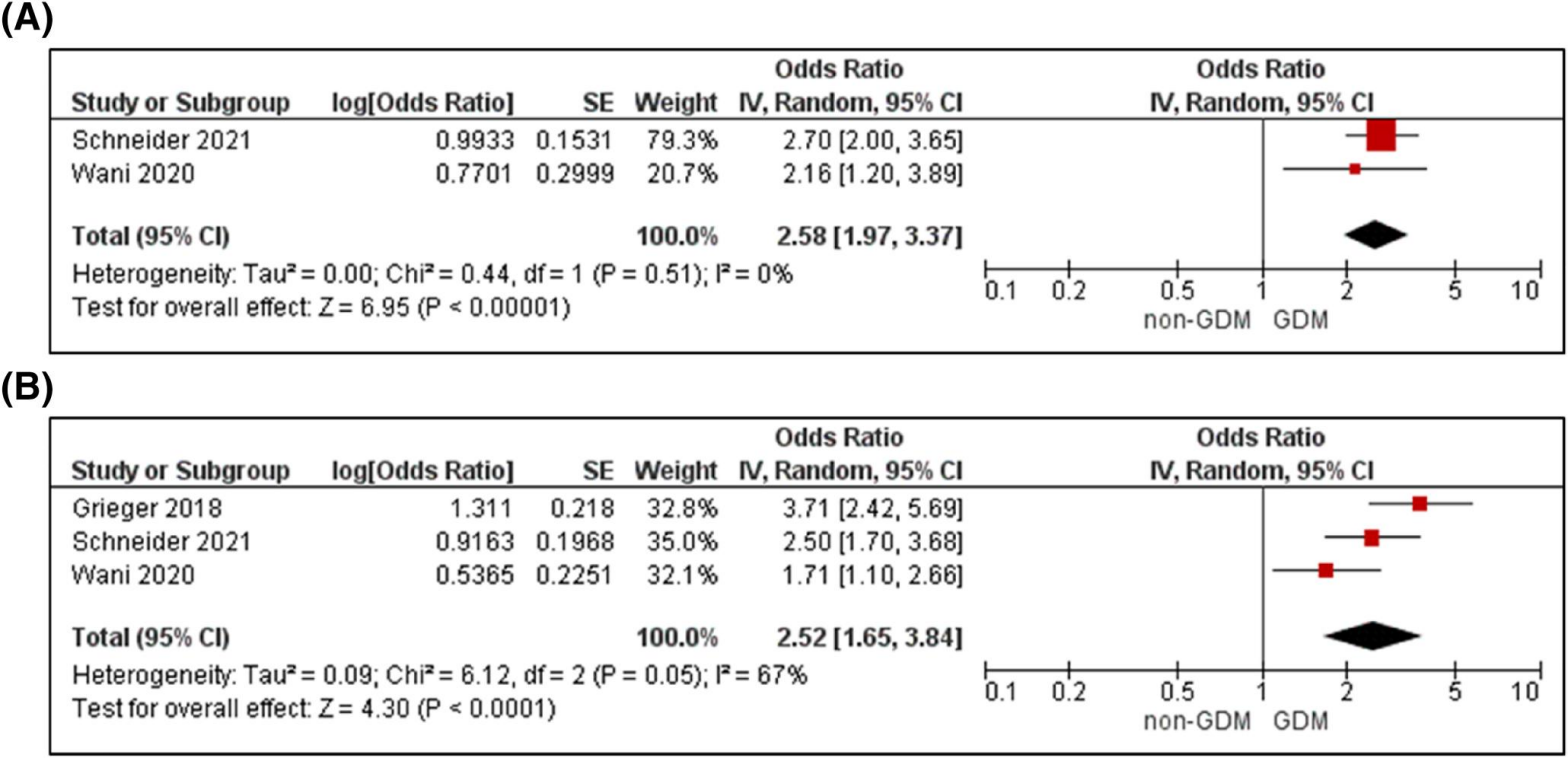
Meta‐analysis of early pregnancy metabolic syndrome and odds of gestational diabetes. Values are odds ratios (OR) with 95% confidence intervals (CIs) for (A) unadjusted and (B) adjusted for maternal age, body mass index (BMI) and family history, analyses. For overall effect, *p*‐value <0.05 was considered significant

Three prospective cohort studies with a sample size ranging from 498 to 3126 were pooled in the meta‐analysis.^24^, ^25^, ^53^ Metabolic syndrome in early pregnancy was associated with a higher odds of GDM in unadjusted (OR 2.58, 95% CI 1.97–3.37, *I* ^*2*^ = 0%, *P*_het_ = 0.51, *k* = 2; Figure 10A) and analyses adjusted for age, BMI and up to another 7 confounders (aOR 2.52, 95% CI 1.65–3.84, *I* ^*2*^ = 67%, *P*_het_ = 0.05, *k* = 3; Figure 10B).

#### 3.3.9 Heterogeneity

Sensitivity analyses were performed on adjusted analyses for BMI (continuous and categorical), FPG, and TG (Supporting Information, Figures S8–S12). Removing the studies with high risk of bias marginally reduced the likelihood of GDM in obese women but did not alter the OR or statistical heterogeneity for the other metabolic factors examined. Excluding the outlier studies with a different direction of effect estimate, the statistical heterogeneity became insignificant for BMI as a continuous variable, and marginally increased the adjusted odds ratio for the effect of TG on GDM. When exploring heterogeneity among the obese BMI category, separately eliminating each study with a large effect estimate did not change the odds of GDM or statistical heterogeneity; however, when removing the three studies together, odds for GDM was reduced, with a moderate, albeit statistically significant change in heterogeneity. There were insufficient numbers of studies to perform sub‐group analyses according to GDM criteria within any metabolic factor (Supporting information Figure S13).

## 4 DISCUSSION

### 4.1 Principal findings

The purpose of this systematic review and meta‐analysis was to examine the association between maternal MetS and its components with GDM, an independent risk factor for future type 2 diabetes and CVD.^6^ Women with overweight or obesity had up to a 4‐fold increased risk for GDM, and increasing FPG or having the MetS as a clustering of factors posed up to a 2.5 times higher likelihood for developing GDM. Findings were consistent in adjusted analyses and persisted in sensitivity analyses to reduce heterogeneity.

### 4.2 Strengths and limitations

Strengths of this review include the extensive and thorough literature search to retrieve relevant eligible studies. The intention to investigate prognostic factors is critically different to prediction models which are used to predict the risk of current disease presence and outcome occurrence in individuals, thereby informing clinical diagnosis. Prognostic research may have an important impact on the translation of interventions from research to clinical practice, to inform health policy and improve patient outcomes. Limitations of the literature reviewed include the overall general moderate or high risk of bias of included studies, mainly because of the lack of adjustment for confounding factors or poor methods of reporting of statistical analyses. The criteria used for diagnosing GDM varied across the included studies, and several studies did not specify how the diagnosis was made. Meta‐analyses also have inherent weaknesses in terms of combining heterogeneous data sets. There was high heterogeneity (*I* ^2^ > 50%) for the pooled adjusted analyses for BMI as a continuous or categorical variable, and also for glucose. However, removing studies with a high risk of bias did not alter the effect estimates or heterogeneity, suggesting study quality does not appear to contribute to an overestimation of the magnitude of the effect that these risk factors have on risk for GDM. Comparatively, removing studies with a different direction of effect or with a larger than usual effect size, reduced heterogeneity indicating publication biases apparent. We could not evaluate clinical heterogeneity from the included studies. It is acknowledged that maternal age, BMI and ethnicity are risk factors for GDM. Yet although the studies in our review enroled younger and older pregnant women or women across the BMI spectrum, the studies did not specifically recruit women who were either younger or older, or with low or high BMI, thus sub‐groups could not be created. While many studies also included women across different ethnic groups, studies did not always report on the proportion of different ethnicities included, and where they did, they were not sufficiently homogenous across studies to make comparisons. Thus, the effect of age, BMI, or ethnicity, on the strength of the association with GDM could not be determined. Nevertheless, we did find that even after adjusting for age and BMI, the effect of the different MetS risk factors on GDM was similar to unadjusted analyses.

### 4.3 Comparison with other studies

Women with overweight or obesity had a 2‐4‐fold greater likelihood for development of GDM. A recent meta‐analysis using 33 observational studies demonstrated up to a 3.2‐fold increased odds for GDM with increasing pre‐pregnancy BMI category, and a 19% increased risk of GDM per unit of increase in pre‐pregnancy BMI.^122^ Our results for early pregnancy overweight or obesity are in line with findings on pre‐pregnancy BMI, albeit, a smaller increase in odds for GDM (8%) per unit increase. Importantly, for our review, we deliberately focussed on early pregnancy BMI, because losing weight before pregnancy does not appear to alter risk for GDM compared to women who are weight stable.^123^, ^124^ Pregnant women with overweight or obesity have higher FPG, insulin, and TG, compared to normal weight pregnant women.^125^ However, several of the individual studies in this review demonstrated that metabolic risk factors increased risk for GDM, independent of BMI. Since weight loss is not recommended during pregnancy,^126^ and targeting pre‐conception women with overweight or obesity is likely to be challenging, our findings reinforce the need to identify other important modifiable risk factors for GDM.

An approximate 2‐fold risk for GDM was demonstrated with increasing level of FPG, which persisted in adjusted and sensitivity analyses. Across gestation, glucose levels reduce due to the maternal adaptations of pregnancy and because of the increased glucose utilisation by the foetal‐placental unit.^127^ There is also an increase in insulin resistance.^128^ These maternal adaptations potentially limit the use of fasting glucose in early pregnancy for early diagnosis of GDM. However, there is data to show that maternal hyperglycaemia before the routine diagnosis of GDM increases the rate of foetal growth^129^ and infant adiposity.^130^, ^131^ Thus whether to diagnose GDM in early pregnancy is an ongoing point of contention. Our results provide some evidence that testing for early FPG may be useful to intervene in women with high glucose to ameliorate the adverse short and long‐term effects of prolonged intrauterine exposure to hyperglycaemia, but the strength of this evidence is insufficient to alter clinical practice or guide timing of early testing. For measurement of HbA1c, a longer‐term measure of glucose control, three studies were included in the meta‐analysis of which one study had a very small OR with large CIs. Thus, whether HbA1c is useful for early screening of future GDM cannot be established from this analysis and requires further investigation.

Increasing fasting TG was associated with a 1.2‐fold increased likelihood for GDM, however from two studies, triglyceride levels >1.7 mmol/L was associated with a 2‐fold increased risk. Increased TG are associated with insulin resistance,^132^ which not only drives the process for MetS,^133^ but is also an important factor underlying the development of type 2 diabetes and CVD.^134^, ^135^, ^136^ In a recent study of 500 adults in China, TG positively correlated with insulin resistance in participants with normal glucose tolerance, with a negative, independent correlation with beta cell function in individuals with dyslipidaemia.^132^ Indeed, a clustering of abnormalities (i.e. metabolic syndrome) which is related to insulin resistance and/or hyperinsulinemia coupled with dyslipidaemia, may be unfavourable to GDM and overall cardiometabolic health. Our systematic review identified only three studies investigating MetS, and pooling of these studies showed a 2.5‐fold higher likelihood for developing GDM. This odds ratio is higher than the individual risk associated with elevated FPG or TG, but lower to that of obesity. While these observations are important and highlight a potentially important relationship between MetS in early pregnancy and risk for GDM, the studies available were few, warranting further investigation.

### 4.4 Recommendations or clinical implications

Overall, our review cannot provide explicit recommendations or implications for practice for women who may benefit early screening and assessment of MetS factors to identify potential risk for GDM. While the effect estimates remained largely unchanged in sensitivity analyses, the overall high heterogeneity could not be sufficiently tested given the available data from the studies. Moreover, sub‐group analyses in populations at higher risk of GDM, such as older maternal age, higher BMI, and women of minority ethnicities, could not be undertaken. The indication that MetS as a cluster of risk factors demonstrated a doubled risk for GDM, warrants further exploration, both in women with or without obesity.

## 5 CONCLUSION

The meta‐analysis provides some evidence that early pregnancy assessment of FPG or the MetS, as a clustering of factors, offers a potential opportunity to detect and treat individual risk factors as an important approach towards GDM prevention. Women with overweight or obesity in pregnancy are also at risk for GDM, however weight loss in pregnancy is not recommended. Given the overall number and quality of studies included, there is a need for further, larger, and higher quality studies to corroborate these results.

## CONFLICT OF INTEREST

None to declare.

## ETHICS STATEMENT

Ethics approval is not applicable because this study is based exclusively on published literature.

## AUTHOR CONTRIBUTIONS

**Nahal Habibi:** Methodology, Formal analysis, Data Curation, Investigation, Writing ‐ Original Draft, Writing ‐ Review & Editing. **Aya Mousa:** Data Curation, Investigation, Writing ‐ Review & Editing, Supervision; Chau Thien Tay: Data Curation, Investigation, Writing ‐ Review & Editing. **Mahnaz Bahri Khomami:** Data Curation, Investigation, Writing ‐ Review & Editing. **Rhiannon K. Patten:** Data Curation, Investigation, Writing ‐ Review & Editing. **Prabha H.  Andraweera:** Data Curation, Investigation, Writing ‐ Review & Editing. **Molla Wassie:** Data Curation, Investigation, Writing ‐ Review & Editing. **Jared Vandersluys:** Data Curation, Investigation, Writing ‐ Review & Editing. **Ali Aflatounian:** Data Curation, Investigation, Writing ‐ Review & Editing. **Tina Bianco‐Miotto:** Conceptualization, Data Curation, Investigation, Writing ‐ Review & Editing. **Shao J. Zhou:** Conceptualization, Data Curation, Investigation, Writing ‐ Review & Editing, Supervision. **Jessica A. Grieger:** Conceptualization, Methodology, Data Curation, Project administration, Funding acquisition, Writing ‐ Review & Editing, Supervision.

### PEER REVIEW

The peer review history for this article is available at https://publons.com/publon/10.1002/dmrr.3532.

## Supporting information

Supplementary Material S1

Supplementary Material S2

Supplementary Material S3

Figure S1

## Data Availability

The data that support the findings of this study are available in All data taken from published studies at https://pubmed.ncbi.nlm.nih.gov. These data were derived from the following resources available in the public domain: Pubmed, https://pubmed.ncbi.nlm.nih.gov.

## References

[1] Nankervis A , Price S , Conn J . Gestational diabetes mellitus: a pragmatic approach to diagnosis and management. Aust J Gen Pract. 2018;47(7):445‐449. 10.31128/AJGP-01-18-4479 30114871

[2] International Diabetes Federation . IDF Diabetes Atlas. In: 9th Edn. Ed. Brussels, Belgium; 2019. https://www.diabetesatlas.org/; https://www.diabetesatlas.org/

[3] Benhalima K , Van Crombrugge P , Moyson C , et al. Characteristics and pregnancy outcomes across gestational diabetes mellitus subtypes based on insulin resistance. Diabetologia. 2019;62(11):2118‐2128. 10.1007/s00125-019-4961-7 31338546

[4] Bryson CL , Ioannou GN , Rulyak SJ , Critchlow C . Association between gestational diabetes and pregnancy‐induced hypertension. Am J Epidemiol. 2003;158(12):1148‐1153. 10.1093/aje/kwg273 14652299

[5] Kim C , Newton KM , Knopp RH . Gestational diabetes and the incidence of type 2 diabetes. A systematic review. 2002;25(10):1862‐1868. 10.2337/diacare.25.10.1862 12351492

[6] Kramer CK , Campbell S , Retnakaran R . Gestational diabetes and the risk of cardiovascular disease in women: a systematic review and meta‐analysis. Diabetologia. 2019;62(6):905‐914. 10.1007/s00125-019-4840-2 30843102

[7] Hapo Study Cooperative Research Group , Metzger BE , Lowe LP, Contreras M, Sacks DA, Watson W, Dooley SL . Hyperglycemia and adverse pregnancy outcomes. N Engl J Med. 2008;358(19):1991‐2002. 10.1056/NEJMoa0707943 18463375

[8] Ornoy A . Prenatal origin of obesity and their complications: gestational diabetes, maternal overweight and the paradoxical effects of fetal growth restriction and macrosomia. Reprod Toxicol. 2011;32(2):205‐212. 10.1016/j.reprotox.2011.05.002 21620955

[9] Young BC , Ecker JL . Fetal macrosomia and shoulder dystocia in women with gestational diabetes: risks amenable to treatment? Curr Diabetes Rep. 2013;13(1):12‐18. 10.1007/s11892-012-0338-8 23076441

[10] Landon MB , Spong CY , Thom E , et al. A multicenter, randomized trial of treatment for mild gestational diabetes. N Engl J Med. 2009;361(14):1339‐1348. 10.1056/NEJMoa0902430 19797280 PMC2804874

[11] Harris DL , Weston PJ , Signal M , Chase JG , Harding JE . Dextrose gel for neonatal hypoglycaemia (the Sugar Babies Study): a randomised, double‐blind, placebo‐controlled trial. Lancet. 2013;382(9910):2077‐2083. 10.1016/S0140-6736(13)61645-1 24075361

[12] Dabelea D . The predisposition to obesity and diabetes in offspring of diabetic mothers. Diabetes Care. 2007;30(Suppl 2):S169‐S174. 10.2337/dc07-s211 17596467

[13] Boney CM , Verma A , Tucker R , Vohr BR . Metabolic syndrome in childhood: association with birth weight, maternal obesity, and gestational diabetes mellitus. Pediatrics. 2005;115(3):e290‐e296. 10.1542/peds.2004-1808 15741354

[14] Rosenberg TJ , Garbers S , Lipkind H , Chiasson MA . Maternal obesity and diabetes as risk factors for adverse pregnancy outcomes: differences among 4 racial/ethnic groups. Am J Publ Health. 2005;95(9):1545‐1551. 10.2105/AJPH.2005.065680 PMC1449396 16118366

[15] Teh WT , Teede HJ , Paul E , Harrison CL , Wallace EM , Allan C . Risk factors for gestational diabetes mellitus: implications for the application of screening guidelines. Aust N Z J Obstet Gynaecol. 2011;51(1):26‐30. 10.1111/j.1479-828X.2011.01292.x 21299505

[16] Yogev Y , Ben‐Haroush A , Chen R , Glickman H , Kaplan B , Hod M . Active induction management of labor for diabetic pregnancies at term; mode of delivery and fetal outcome—a single center experience. Eur J Obstet Gynecol Reprod Biol. 2004;114(2):166‐170. 10.1016/j.ejogrb.2003.10.017 15140510

[17] Muche AA , Olayemi OO , Gete YK . Prevalence and determinants of gestational diabetes mellitus in Africa based on the updated international diagnostic criteria: a systematic review and meta‐analysis. Arch Public Health. 2019;77(1):36. 10.1186/s13690-019-0362-0 31402976 PMC6683510

[18] Bhattacharya SM . Fasting or two‐hour postprandial plasma glucose levels in early months of pregnancy as screening tools for gestational diabetes mellitus developing in later months of pregnancy. J Obstet Gynaecol Res. 2004;30(4):333‐336. 10.1111/j.1447-0756.2004.00205.x 15238113

[19] Agarwal MM , Dhatt GS , Punnose J , Zayed R . Gestational diabetes: fasting and postprandial glucose as first prenatal screening tests in a high‐risk population. J Reprod Med. 2007;52(4):299‐305. 17506370

[20] Sacks DA , Chen W , Wolde‐Tsadik G , Buchanan TA . Fasting plasma glucose test at the first prenatal visit as a screen for gestational diabetes. Obstet Gynecol. 2003;101(6):1197‐1203. 10.1016/s0029-7844(03)00049-8 12798525

[21] Phillips CM . Metabolically healthy obesity: definitions, determinants and clinical implications. Rev Endocr Metabol Disord. 2013;14(3):219‐227. 10.1007/s11154-013-9252-x 23928851

[22] Fan J , Song Y , Chen Y , Hui R , Zhang W . Combined effect of obesity and cardio‐metabolic abnormality on the risk of cardiovascular disease: a meta‐analysis of prospective cohort studies. Int J Cardiol. 2013;168(5):4761‐4768. 10.1016/j.ijcard.2013.07.230 23972953

[23] Ford ES , Li C , Sattar N . Metabolic syndrome and incident diabetes: current state of the evidence. Diabetes Care. 2008;31(9):1898‐1904. 10.2337/dc08-0423 18591398 PMC2518368

[24] Grieger JA , Bianco‐Miotto T , Grzeskowiak LE , et al. Metabolic syndrome in pregnancy and risk for adverse pregnancy outcomes: a prospective cohort of nulliparous women. PLoS Med. 2018;15(12):e1002710. 10.1371/journal.pmed.1002710 30513077 PMC6279018

[25] Schneider AK , Leemaqz SY , Dalton J , et al. The interaction between metabolic syndrome and physical activity, and risk for gestational diabetes mellitus. Acta Diabetol. 2021;58(7):939‐947. 10.1007/s00592-021-01696-9 33743081

[26] Barrett HL , Dekker Nitert M , McIntyre HD , Callaway LK . Normalizing metabolism in diabetic pregnancy: is it time to target lipids? Diabetes Care. 2014;37(5):1484‐1493. 10.2337/dc13-1934 24757231

[27] Moayeri M , Heida KY , Franx A , Spiering W , de Laat MW , Oudijk MA . Maternal lipid profile and the relation with spontaneous preterm delivery: a systematic review. Arch Gynecol Obstet. 2017;295(2):313‐323. 10.1007/s00404-016-4216-5 27807624 PMC5281656

[28] Ryckman KK , Spracklen CN , Smith CJ , Robinson JG , Saftlas AF . Maternal lipid levels during pregnancy and gestational diabetes: a systematic review and meta‐analysis. BJOG. 2015;122(5):643‐651. 10.1111/1471-0528.13261 25612005

[29] Thom G , Lean M . Is there an optimal diet for weight management and metabolic health? Gastroenterology. 2017;152(7):1739‐1751. 10.1053/j.gastro.2017.01.056 28214525

[30] Wang Y , Xu D . Effects of aerobic exercise on lipids and lipoproteins. Lipids Health Dis. 2017;16(1):132. 10.1186/s12944-017-0515-5 28679436 PMC5498979

[31] Low Wang CC , Hess CN , Hiatt WR , Goldfine AB . Clinical update: cardiovascular disease in diabetes mellitus: atherosclerotic cardiovascular disease and heart failure in type 2 diabetes mellitus—mechanisms, management, and clinical considerations. Circulation. 2016;133(24):2459‐2502. 10.1161/circulationaha.116.022194 27297342 PMC4910510

[32] Donovan BM , Nidey NL , Jasper EA , et al. First trimester prenatal screening biomarkers and gestational diabetes mellitus: a systematic review and meta‐analysis. PLoS One. 2018;13(7):e0201319. 10.1371/journal.pone.0201319 30048548 PMC6062092

[33] Yao D , Chang Q , Wu Q‐J , et al. Relationship between maternal central obesity and the risk of gestational diabetes mellitus: a systematic review and meta‐analysis of cohort studies. J Diabetes Res. 2020;2020:6303820. 10.1155/2020/6303820 32337296 PMC7157762

[34] Lamain‐de Ruiter M , Kwee A , Naaktgeboren CA , Franx A , Moons KGM , Koster MPH . Prediction models for the risk of gestational diabetes: a systematic review. Diagn Prognostic Res. 2017;1:3. 10.1186/s41512-016-0005-7 PMC6457144 31093535

[35] Lorenzo‐Almorós A , Hang T , Peiró C , et al. Predictive and diagnostic biomarkers for gestational diabetes and its associated metabolic and cardiovascular diseases. Cardiovasc Diabetol. 2019;18(1):140. 10.1186/s12933-019-0935-9 31666083 PMC6820966

[36] Shepherd E , Gomersall JC , Tieu J , Han S , Crowther CA , Middleton P . Combined diet and exercise interventions for preventing gestational diabetes mellitus. Cochrane Database Syst Rev. 2017(11). 10.1002/14651858.CD010443.pub3 PMC6485974 29129039

[37] Page MJ , McKenzie JE , Bossuyt PM , et al. The PRISMA 2020 statement: an updated guideline for reporting systematic reviews. BMJ Clin Res ed. 2021;372:n71. 10.1136/bmj.n71 PMC8005924 33782057

[38] Geersing GJ , Bouwmeester W , Zuithoff P , Spijker R , Leeflang M , Moons KG . Search filters for finding prognostic and diagnostic prediction studies in Medline to enhance systematic reviews. PLoS One. 2012;7(2):e32844. 10.1371/journal.pone.0032844 22393453 PMC3290602

[39] Alberti KGMM , Zimmet P , Shaw J . Metabolic syndrome—a new world‐wide definition. A consensus statement from the International Diabetes Federation. Diabet Med. 2006;23(5):469‐480. 10.1111/j.1464-5491.2006.01858.x 16681555

[40] Grundy SM , Cleeman JI , Daniels SR , et al. Diagnosis and management of the metabolic syndrome: an American Heart Association/National Heart, Lung, and Blood Institute Scientific Statement. Circulation. 2005;112(17):2735‐2752. 10.1161/circulationaha.105.169404 16157765

[41] Cavero‐Redondo I , Martínez‐Vizcaíno V , Álvarez‐Bueno C , Agudo‐Conde C , Lugones‐Sánchez C , García‐Ortiz L . Metabolic syndrome including glycated hemoglobin A1c in adults: is it time to change? J Clin Med. 2019;8(12):2090. 10.3390/jcm8122090 PMC6947260 31805696

[42] Ouzzani M , Hammady H , Fedorowicz Z , Elmagarmid A . Rayyan‐a web and mobile app for systematic reviews. Syst Rev. 2016;5(1):210. 10.1186/s13643-016-0384-4 27919275 PMC5139140

[43] Riley RD , Moons KGM , Snell KIE , et al. A guide to systematic review and meta‐analysis of prognostic factor studies. BMJ. 2019;364:k4597. 10.1136/bmj.k4597 30700442

[44] Langan D , Higgins JPT , Jackson D , et al. A comparison of heterogeneity variance estimators in simulated random‐effects meta‐analyses. Res Synth methods. 2019;10(1):83‐98. 10.1002/jrsm.1316 30067315

[45] Higgins JPT , Thompson SG , Deeks JJ , Altman DG . Measuring inconsistency in meta‐analyses. BMJ. 2003;327(7414):557‐560. 10.1136/bmj.327.7414.557 12958120 PMC192859

[46] Patsopoulos NA , Evangelou E , Ioannidis JP . Sensitivity of between‐study heterogeneity in meta‐analysis: proposed metrics and empirical evaluation. Int J Epidemiol. 2008;37(5):1148‐1157. 10.1093/ije/dyn065 18424475 PMC6281381

[47] Sterne JA , Gavaghan D , Egger M . Publication and related bias in meta‐analysis: power of statistical tests and prevalence in the literature. J Clin Epidemiol. 2000;53(11):1119‐1129. 10.1016/s0895-4356(00)00242-0 11106885

[48] Sánchez‐Vera I , Bonet B , Viana M , et al. Changes in plasma lipids and increased low‐density lipoprotein susceptibility to oxidation in pregnancies complicated by gestational diabetes: consequences of obesity. Metabolism. 2007;56(11):1527‐1533. 10.1016/j.metabol.2007.06.020 17950104

[49] Doi L , Williams AJ , Marryat L , Frank J . Cohort study of high maternal body mass index and the risk of adverse pregnancy and delivery outcomes in Scotland. BMJ Open. 2020;10(2):e026168. 10.1136/bmjopen-2018-026168 PMC7045241 32086347

[50] International association of diabetes and pregnancy study groups recommendations on the diagnosis and classification of hyperglycemia in pregnancy. Diabetes Care. 2010;33(3):676‐682, 10.2337/dc09-1848 20190296 PMC2827530

[51] World Health Organization . Definition, Diagnosis and Classification of Diabetes Mellitus and its Complications. Part 1: Diagnosis and Classification of Diabetes Mellitus; 1999. https://apps.who.int/iris/handle/10665/66040

[52] Carpenter MW , Coustan DR . Criteria for screening tests for gestational diabetes. Am J Obstet Gynecol. 1982;144(7):768‐773. 10.1016/0002-9378(82)90349-0 7148898

[53] Wani K , Sabico S , Alnaami AM , et al. Early‐pregnancy metabolic syndrome and subsequent incidence in gestational diabetes mellitus in Arab women. Front Endocrinol (Lausanne). 2020;11:98. 10.3389/fendo.2020.00098 32174891 PMC7056831

[54] Zhu Y , Hedderson MM , Quesenberry CP , Feng J , Ferrara A . Central obesity increases the risk of gestational diabetes partially through increasing insulin resistance. Obes (Silver Spring). 2019;27(1):152‐160. 10.1002/oby.22339 PMC6309219 30461219

[55] Han Q , Shao P , Leng J , et al. Interactions between general and central obesity in predicting gestational diabetes mellitus in Chinese pregnant women: a prospective population‐based study in Tianjin, China. J Diabetes. 2018;10(1):59‐67. 10.1111/1753-0407.12558 28383185

[56] Hancerliogullari N , Kansu‐Celik H , Asli Oskovi‐Kaplan Z , Kisa B , Engin‐Ustun Y , Ozgu‐Erdinc AS . Optimal maternal neck and waist circumference cutoff values for prediction of gestational diabetes mellitus at the first trimester in Turkish population; a prospective cohort study. Gynecol Endocrinol. 2020;36(11):1002‐1005. 10.1080/09513590.2020.1750003 32274939

[57] Alptekin H , Çizmecioğlu A , Işık H , Cengiz T , Yildiz M , Iyisoy MS . Predicting gestational diabetes mellitus during the first trimester using anthropometric measurements and HOMA‐IR. J Endocrinol Invest. 2016;39(5):577‐583. 10.1007/s40618-015-0427-z 26754418

[58] Gao S , Leng J , Liu H , et al. Development and validation of an early pregnancy risk score for the prediction of gestational diabetes mellitus in Chinese pregnant women. BMJ Open Diabetes Res Care. 2020;8(1):e000909. 10.1136/bmjdrc-2019-000909 PMC7202751 32327440

[59] Basraon SK , Mele L , Myatt L , et al. Relationship of early pregnancy waist‐to‐hip ratio versus body mass index with gestational diabetes mellitus and insulin resistance. Am J Perinatol. 2016;33(1):114‐121. 10.1055/s-0035-1562928 26352680 PMC5283057

[60] Falcone V , Kotzaeridi G , Breil MH , et al. Early assessment of the risk for gestational diabetes mellitus: can fasting parameters of glucose metabolism contribute to risk prediction? Diabetes Metab J. 2019;43(6):785‐793. 10.4093/dmj.2018.0218 30877716 PMC6943268

[61] Kumru P , Arisoy R , Erdogdu E , et al. Prediction of gestational diabetes mellitus at first trimester in low‐risk pregnancies. Taiwan J Obstet Gynecol. 2016;55(6):815‐820. 10.1016/j.tjog.2016.04.032 28040126

[62] Li P , Lin S , Li L , Cui J , Zhou S , Fan J . First‐trimester fasting plasma glucose as a predictor of gestational diabetes mellitus and the association with adverse pregnancy outcomes. Pak J Med Sci. 2019;35(1):95‐100. 10.12669/pjms.35.1.216 30881404 PMC6408635

[63] Liu PJ , Liu Y , Ma L , et al. The predictive ability of two triglyceride‐associated indices for gestational diabetes mellitus and large for gestational age infant among Chinese pregnancies: a preliminary cohort study. Diabetes Metab Syndr Obes. 2020;13:2025‐2035. 10.2147/dmso.s251846 32606861 PMC7305827

[64] Li G , Huang W , Zhang L , et al. A prospective cohort study of early‐pregnancy risk factors for gestational diabetes in polycystic ovarian syndrome. Diabetes/Metabol Res Rev. 2018;34(5):e3003. 10.1002/dmrr.3003 29514404

[65] Pazhohan A , Rezaee Moradali M , Pazhohan N . Association of first‐trimester maternal lipid profiles and triglyceride‐glucose index with the risk of gestational diabetes mellitus and large for gestational age newborn. J Matern Fetal Neonatal Med. 2019;32(7):1167‐1175. 10.1080/14767058.2017.1402876 29157043

[66] Savvidou M , Nelson SM , Makgoba M , Messow CM , Sattar N , Nicolaides K . First‐trimester prediction of gestational diabetes mellitus: examining the potential of combining maternal characteristics and laboratory measures. Diabetes. 2010;59(12):3017‐3022. 10.2337/db10-0688 20876721 PMC2992761

[67] Nanda S , Savvidou M , Syngelaki A , Akolekar R , Nicolaides KH . Prediction of gestational diabetes mellitus by maternal factors and biomarkers at 11 to 13 weeks. Prenat Diagn. 2011;31(2):135‐141. 10.1002/pd.2636 21268030

[68] Syngelaki A , Bredaki FE , Vaikousi E , Maiz N , Nicolaides KH . Body mass index at 11‐13 weeks' gestation and pregnancy complications. Fetal Diagn Ther. 2011;30(4):250‐265. 10.1159/000328083 22067258

[69] Gabbay‐Benziv R , Doyle LE , Blitzer M , Baschat AA . First trimester prediction of maternal glycemic status. J Perinat Med. 2015;43(3):283‐289. 10.1515/jpm-2014-0149 25153547

[70] Sánchez‐García A , Rodríguez‐Gutiérrez R , Saldívar‐Rodríguez D , et al. Early triglyceride and glucose index as a risk marker for gestational diabetes mellitus. Int J Gynaecol Obstet. 2020;171(1):117‐123. 10.1002/ijgo.13311 32679624

[71] Sweeting AN , Appelblom H , Ross GP , et al. First trimester prediction of gestational diabetes mellitus: a clinical model based on maternal demographic parameters. Diabetes Res Clin Pract. 2017;127:44‐50. 10.1016/j.diabres.2017.02.036 28319801

[72] Riskin‐Mashiah S , Damti A , Younes G , Auslender R . First trimester fasting hyperglycemia as a predictor for the development of gestational diabetes mellitus. Eur J Obstet Gynecol Reprod Biol. 2010;152(2):163‐167. 10.1016/j.ejogrb.2010.05.036 20579799

[73] Yachi Y , Tanaka Y , Anasako Y , Nishibata I , Saito K , Sone H . Contribution of first trimester fasting plasma insulin levels to the incidence of glucose intolerance in later pregnancy: Tanaka Women's Clinic Study. Diabetes Res Clin Pract. 2011;92(2):293‐298. 10.1016/j.diabres.2011.02.012 21396732

[74] Migda M , Migda MS , Migda B , Krzyżanowska P , Wender‐Ożegowska E . Components of metabolic syndrome in the first trimester of pregnancy as predictors of adverse perinatal outcome. Ginekol Pol. 2016;87(9):644‐650. 10.5603/gp.2016.0060 27723072

[75] Cozzolino M , Serena C , Maggio L , et al. Analysis of the main risk factors for gestational diabetes diagnosed with International Association of Diabetes and Pregnancy Study Groups (IADPSG) criteria in multiple pregnancies. J Endocrinol Invest. 2017;40(9):937‐943. 10.1007/s40618-017-0646-6 28324453

[76] Farah N , McGoldrick A , Fattah C , O'Connor N , Kennelly MM , Turner MJ . Body mass index (BMI) and glucose intolerance during pregnancy in white European women. J Reproduction Infertil. 2012;13(2):95‐99. PMC3719339 23926531

[77] Teede HJ , Harrison CL , Teh WT , Paul E , Allan CA . Gestational diabetes: development of an early risk prediction tool to facilitate opportunities for prevention. Aust N Z J Obstet Gynaecol. 2011;51(6):499‐504. 10.1111/j.1479-828X.2011.01356.x 21951203

[78] Collier A , Abraham EC , Armstrong J , Godwin J , Monteath K , Lindsay R . Reported prevalence of gestational diabetes in Scotland: the relationship with obesity, age, socioeconomic status, smoking and macrosomia, and how many are we missing? J Diabetes Investig. 2017;8(2):161‐167. 10.1111/jdi.12552 PMC5334332 27397133

[79] Knight‐Agarwal CR , Williams LT , Davis D , et al. Association of BMI and interpregnancy BMI change with birth outcomes in an Australian obstetric population: a retrospective cohort study. BMJ Open. 2016;10(6):5. 10.1136/bmjopen-2015-010667 PMC4874127 27165646

[80] Raja UA , McAree T , Bassett P , Sharma S . The implications of a raised maternal BMI: a DGH experience. J Obstet Gynaecol. 2012;32(3):247‐251. 10.3109/01443615.2011.645920 22369397

[81] El‐Gilany AH , Hammad S . Body mass index and obstetric outcomes in pregnant in Saudi Arabia: a prospective cohort study. Ann Saudi Med. 2010;30(5):376‐380. 10.4103/0256-4947.67075 20697173 PMC2941250

[82] Schrauwers C , Dekker G . Maternal and perinatal outcome in obese pregnant patients. J Matern Fetal Neonatal Med. 2009;22(3):218‐226. 10.1080/14767050902801652 19330705

[83] Phaloprakarn C , Tangjitgamol S , Manusirivithaya S . A risk score for selective screening for gestational diabetes mellitus. Eur J Obstet Gynecol Reprod Biol. 2009;145(1):71‐75. 10.1016/j.ejogrb.2009.04.016 19446948

[84] Denison F , Norwood P , Bhattacharya S , et al. Association between maternal body mass index during pregnancy, short‐term morbidity, and increased health service costs: a population‐based study. BJOG Int J Obstet Gynaecol. 2014;121(1):72‐82. 10.1111/1471-0528.12443 24102880

[85] Lei Q , Niu J , Lv L , et al. Clustering of metabolic risk factors and adverse pregnancy outcomes: a prospective cohort study. Diabetes/Metabol Res Rev. 2016;32(8):835‐842. 10.1002/dmrr.2803 27037671

[86] Magann EF , Doherty DA , lin AT , Chauhan SP , Morrison JC . The effects of an increasing gradient of maternal obesity on pregnancy outcomes. Aust N Z J Obstet Gynaecol. 2013;53(3):250‐257. 10.1111/ajo.12047 23432797

[87] Simko M , Totka A , Vondrova D , et al. Maternal body mass index and gestational weight gain and their association with pregnancy complications and perinatal conditions. Int J Environ Res Publ Health. 2019;16(10):1751. 10.3390/ijerph16101751 PMC6572546 31108864

[88] Zhao YN , Li Q , Li YC . Effects of body mass index and body fat percentage on gestational complications and outcomes. J Obstet Gynaecol Res. 2014;40(3):705‐710. 10.1111/jog.12240 24738116

[89] Hashemi‐Nazari S‐S , Najafi F , Rahimi M‐A , Izadi N , Heydarpour F , Forooghirad H . Estimation of gestational diabetes mellitus and dose–response association of BMI with the occurrence of diabetes mellitus in pregnant women of the west of Iran. Health Care Women Int. 2020;41(1):121‐130. 10.1080/07399332.2018.1521812 30433854

[90] Sreedevi C , Valsaraj BP , Pais M . A correlative study to assess the effect of first trimester BMI on obstetric outcome. Int J Nurs Educ Scholarsh. 2012;4(1):35‐36.

[91] Yang Z , Phung H , Freebairn L , Sexton R , Raulli A , Kelly P . Contribution of maternal overweight and obesity to the occurrence of adverse pregnancy outcomes. Aust N Z J Obstet Gynaecol. 2019;59(3):367‐374. 10.1111/ajo.12866 30024043

[92] Zheng W , Huang W , Zhang L , et al. Early pregnancy metabolic factors associated with gestational diabetes mellitus in normal‐weight women with polycystic ovary syndrome: a two‐phase cohort study. Diabetol Metab Syndrome. 2019;11(1):71. 10.1186/s13098-019-0462-6 PMC6708128 31462934

[93] Berggren EK , Boggess KA , Mathew L , Culhane J . First trimester maternal glycated hemoglobin and sex hormone‐binding globulin do not predict third trimester glucose intolerance of pregnancy. Reprod Sci. 2017;24(4):613‐618. 10.1177/1933719116667230 27613817

[94] Iyoke CA , Ugwu GO , Ezugwu FO , Lawani OL , Onyebuchi AK . Retrospective cohort study of the effects of obesity in early pregnancy on maternal weight gain and obstetric outcomes in an obstetric population in Africa. Int J Womens Health. 2013;5:501‐507. 10.2147/ijwh.s49909 23983492 PMC3747850

[95] Madhavan A , Beena Kumari R , Sanal MG . A pilot study on the usefulness of body mass index and waist hip ratio as a predictive tool for gestational diabetes in Asian Indians. Gynecol Endocrinol. 2008;24(12):701‐707. 10.1080/09513590802444134 19172540

[96] Wolfe HM , Zador IE , Gross TL , Martier SS , Sokol RJ . The clinical utility of maternal body mass index in pregnancy. Am J Obstet Gynecol. 1991;164(5 Pt 1):1306‐1310. 10.1016/0002-9378(91)90705-v 2035574

[97] Ozgu‐Erdinc AS , Yilmaz S , Yeral MI , Seckin KD , Erkaya S , Danisman AN . Prediction of gestational diabetes mellitus in the first trimester: comparison of C‐reactive protein, fasting plasma glucose, insulin and insulin sensitivity indices. J Matern Fetal Neonatal Med. 2015;28(16):1957‐1962. 10.3109/14767058.2014.973397 25283990

[98] Al‐Shafei AI , Rayis DA , Mohieldein AH , El‐Gendy OA , Adam I . Maternal early pregnancy serum level of 25‐Hydroxyvitamin D and risk of gestational diabetes mellitus. Int J Gynecol Obstet. 2021;152(3):382‐385. 10.1002/ijgo.13389 32976628

[99] Leng J , Shao P , Zhang C , et al. Prevalence of gestational diabetes mellitus and its risk factors in Chinese pregnant women: a prospective population‐based study in Tianjin, China. PLoS One. 2015;10(3):e0121029. 10.1371/journal.pone.0121029 25799433 PMC4370728

[100] Tenenbaum‐Gavish K , Sharabi‐Nov A , Binyamin D , et al. First trimester biomarkers for prediction of gestational diabetes mellitus. Placenta. 2020;101:80‐89. 10.1016/j.placenta.2020.08.020 32937245

[101] Zhang X , Zhao X , Huo L , et al. Risk prediction model of gestational diabetes mellitus based on nomogram in a Chinese population cohort study. Sci Rep. 2020;10(1):21223. 10.1038/s41598-020-78164-x 33277541 PMC7718223

[102] Kouhkan A , Khamseh ME , Moini A , et al. Predictive factors of gestational diabetes in pregnancies following assisted reproductive technology: a nested case–control study. Arch Gynecol Obstet. 2018;298(1):199‐206. 10.1007/s00404-018-4772-y 29730813

[103] Godwin M , Muirhead M , Huynh J , Helt B , Grimmer J . Prevalence of gestational diabetes mellitus among Swampy Cree women in moose factory, James Bay. CMAJ (Can Med Assoc J). 1999;160(9):1299‐1302. 10333831 PMC1230311

[104] Guo F , Yang S , Zhang Y , Yang X , Zhang C , Fan J . Nomogram for prediction of gestational diabetes mellitus in urban, Chinese, pregnant women. BMC Pregnancy Childbirth. 2020;20(43). 10.1186/s12884-019-2703-y PMC6971941 31959134

[105] Meek CL , Lindsay RS , Scott EM , et al. Approaches to screening for hyperglycaemia in pregnant women during and after the COVID‐19 pandemic. Diabet Med. 2021;38(1):e14380. 10.1111/dme.14380 32750184 PMC7436759

[106] Kansu‐Celik H , Ozgu‐Erdinc AS , Kisa B , Eldem S , Hancerliogullari N , Engin‐Ustun Y . Maternal serum glycosylated hemoglobin and fasting plasma glucose predicts gestational diabetes at the first trimester in Turkish women with a low‐risk pregnancy and its relationship with fetal birth weight; a retrospective cohort study. J Matern Fetal Neonatal Med. 2019;12:1‐8. 10.1080/14767058.2019.1651837 31370710

[107] Wang C , Zhu W , Wei Y , et al. The predictive effects of early pregnancy lipid profiles and fasting glucose on the risk of gestational diabetes mellitus stratified by body mass index. J Diabetes Res. 2016;2016:3013567. 10.1155/2016/3013567 26981541 PMC4770134

[108] Gur EB , Ince O , Turan GA , et al. Ultrasonographic visceral fat thickness in the first trimester can predict metabolic syndrome and gestational diabetes mellitus. Endocrine. 2014;47(2):478‐484. 10.1007/s12020-013-0154-1 24452873

[109] Zhu WW , Yang HX , Wei YM , et al. Evaluation of the value of fasting plasma glucose in the first prenatal visit to diagnose gestational diabetes mellitus in China. Diabetes Care. 2013;36(3):586‐590. 10.2337/dc12-1157 23193214 PMC3579369

[110] Sesmilo G , Prats P , Garcia S , et al. First‐trimester fasting glycemia as a predictor of gestational diabetes (GDM) and adverse pregnancy outcomes. Acta Diabetol. 2019;57(6):697‐703. 10.12669/pjms.35.1.216 31984438

[111] Ogonowski J , Miazgowski T , Homa K , Celewicz K , Kuczyńska M . Low predictive value of traditional risk factors in identifying women at risk for gestational diabetes. Acta Obstet Gynecol Scand. 2007;86(10):1162‐1170. 10.1080/00016340701505044 17851799

[112] Amylidi S , Mosimann B , Stettler C , Fiedler GM , Surbek D , Raio L . First‐trimester glycosylated hemoglobin in women at high risk for gestational diabetes. Acta Obstet Gynecol Scand. 2016;95(1):93‐97. 10.1111/aogs.12784 26400192

[113] Arbib N , Shmueli A , Salman L , Krispin E , Toledano Y , Hadar E . First trimester glycosylated hemoglobin as a predictor of gestational diabetes mellitus. Int J Gynaecol Obstet. 2019;145(2):158‐163. 10.1002/ijgo.12794 30791100

[114] Punnose J , Malhotra RK , Sukhija K , Mathew A , Sharma A , Choudhary N . Glycated haemoglobin in the first trimester: a predictor of gestational diabetes mellitus in pregnant Asian Indian women. Diabetes Res Clin Pract. 2020;159:107953. 10.1016/j.diabres.2019.107953 31794807

[115] Hinkle SN , Tsai MY , Rawal S , Albert PS , Zhang C . HbA(1c) measured in the first trimester of pregnancy and the association with gestational diabetes. Sci Rep. 2018;8(1):12249. 10.1038/s41598-018-30833-8 30116010 PMC6095876

[116] Odsæter IH , Åsberg A , Vanky E , Carlsen SM . HbA1c as screening for gestational diabetes mellitus in women with polycystic ovary syndrome. BMC Endocr Disord. 2015;15(1):38. 10.1186/s12902-015-0039-9 26245653 PMC4527320

[117] Wang C , Zhu W , Wei Y , et al. The associations between early pregnancy lipid profiles and pregnancy outcomes. J Perinatol. 2017;37(2):127‐133. 10.1038/jp.2016.191 27787507

[118] Wen‐Yuan J , Sheng‐Liang L , Ruo‐Lin H , et al. Associations between maternal lipid profile and pregnancy complications and perinatal outcomes: a population‐based study from China. BMC Pregnancy Childbirth. 2016;16:1‐9. 10.1186/s12884-016-0852-9 27000102 PMC4802610

[119] Zhang Y , Lan X , Cai C , et al. Associations between maternal lipid profiles and pregnancy complications: a prospective population‐based study. Am J Perinatol. 2019;38(08):834‐840. 10.1055/s-0039-3402724 31891957

[120] Bao W , Dar S , Zhu Y , et al. Plasma concentrations of lipids during pregnancy and the risk of gestational diabetes mellitus: a longitudinal study. J Diabetes. 2018;10(6):487‐495. 10.1111/1753-0407.12563 28436169 PMC5837900

[121] Zhu H , He D , Liang N , Lai A , Zeng J , Yu H . High serum triglyceride levels in the early first trimester of pregnancy are associated with gestational diabetes mellitus: a prospective cohort study. J Diabetes Investig. 2020;11(6):1635‐1642. 10.1111/jdi.13273 PMC7610113 32281298

[122] Najafi F , Hasani J , Izadi N , et al. The effect of prepregnancy body mass index on the risk of gestational diabetes mellitus: a systematic review and dose‐response meta‐analysis. Obes Rev. 2019;20(3):472‐486. 10.1111/obr.12803 30536891

[123] Hedderson MM , Williams MA , Holt VL , Weiss NS , Ferrara A . Body mass index and weight gain prior to pregnancy and risk of gestational diabetes mellitus. Am J Obstet Gynecol. 2008;198(4):409.e1‐409.e7. 10.1016/j.ajog.2007.09.028 18068138 PMC2696228

[124] Adane AA , Tooth LR , Mishra GD . Pre‐pregnancy weight change and incidence of gestational diabetes mellitus: a finding from a prospective cohort study. Diabetes Res Clin Pract. 2017;124:72‐80. 10.1016/j.diabres.2016.12.014 28110238

[125] Roland MCP , Lekva T , Godang K , Bollerslev J , Henriksen T . Changes in maternal blood glucose and lipid concentrations during pregnancy differ by maternal body mass index and are related to birthweight: a prospective, longitudinal study of healthy pregnancies. PLoS One. 2020;15(6):e0232749. 10.1371/journal.pone.0232749 32574162 PMC7310681

[126] Kapadia MZ , Park CK , Beyene J , Giglia L , Maxwell C , McDonald SD . Weight loss instead of weight gain within the guidelines in obese women during pregnancy: a systematic review and meta‐analyses of maternal and infant outcomes. PLoS One. 2015;10(7):e0132650. 10.1371/journal.pone.0132650 26196130 PMC4509670

[127] Di Cianni G , Miccoli R , Volpe L , Lencioni C , Del Prato S . Intermediate metabolism in normal pregnancy and in gestational diabetes. Diabetes Metab Res Rev. 2003;19(4):259‐270. 10.1002/dmrr.390 12879403

[128] Newbern D , Freemark M . Placental hormones and the control of maternal metabolism and fetal growth. Curr Opin Endocrinol diabetes, Obes. 2011;18(6):409‐416. 10.1097/MED.0b013e32834c800d 21986512

[129] Sovio U , Murphy HR , Smith GC . Accelerated fetal growth prior to diagnosis of gestational diabetes mellitus: a prospective cohort study of nulliparous women. Diabetes Care. 2016;39(6):982‐987. 10.2337/dc16-0160 27208333

[130] Logan KM , Emsley RJ , Jeffries S , et al. Development of early adiposity in infants of mothers with gestational diabetes mellitus. Diabetes Care. 2016;39(6):1045‐1051. 10.2337/dc16-0030 27208326

[131] Sweeting AN , Ross GP , Hyett J , Wong J . Gestational diabetes in the first trimester: is early testing justified? lancet Diabetes & Endocrinol. 2017;5(8):571‐573. 10.1016/s2213-8587(17)30066-9 28258842

[132] Ma M , Liu H , Yu J , et al. Triglyceride is independently correlated with insulin resistance and islet beta cell function: a study in population with different glucose and lipid metabolism states. Lipids Health Dis. 2020;19(1):121. 10.1186/s12944-020-01303-w 32487177 PMC7268278

[133] Haffner SM , Stern MP , Hazuda HP , Mitchell BD , Patterson JK . Cardiovascular risk factors in confirmed prediabetic individuals. Does the clock for coronary heart disease start ticking before the onset of clinical diabetes? JAMA. 1990;263(21):2893‐2898. 10.1001/jama.263.21.2893 2338751

[134] Laakso M , Kuusisto J . Insulin resistance and hyperglycaemia in cardiovascular disease development. Nat Rev Endocrinol. 2014;10(5):293‐302. 10.1038/nrendo.2014.29 24663222

[135] Vergès B . Pathophysiology of diabetic dyslipidaemia: where are we? Diabetologia. 2015;58(5):886‐899. 10.1007/s00125-015-3525-8 25725623 PMC4392164

[136] Abdul‐Ghani MA , Tripathy D , DeFronzo RA . Contributions of β‐cell dysfunction and insulin resistance to the pathogenesis of impaired glucose tolerance and impaired fasting glucose. Diabetes Care. 2006;29(5):1130‐1139. 10.2337/dc05-2179 16644654

