# Supplementary material for: Maternal metabolic factors and the association with gestational diabetes: A systematic review and meta‐analysis
Source: Diabetes Metab Res Rev. 2022 Apr 25;38(5):e3532. doi: 10.1002/dmrr.3532 (PMC9540632; doi:10.1002/dmrr.3532)
Supplement: Supplementary file 1 — Supplementary Material S1 [file DMRR-38-e3532-s004.docx]

**Supporting Information: Search strategy**

**PUBMED**

((((((((((("pregnant women"[MeSH Terms] OR "maternal health"[MeSH Terms]) OR "pregnan*"[Title/Abstract]) OR "gestat*"[Title/Abstract]) OR "first trimester"[Title/Abstract]) AND (((((((((((((((((("cholesterol"[Title/Abstract] OR "glucose"[Title/Abstract]) OR "body mass index"[Title/Abstract]) OR "lipids"[Title/Abstract]) OR "hdl"[Title/Abstract]) OR "high density lipoprotein cholesterol"[Title/Abstract]) OR "triglyceride"[Title/Abstract]) OR "triacylglycerol"[Title/Abstract]) OR "blood pressure"[Title/Abstract]) OR "waist circumference"[Title/Abstract]) OR "central obesity"[Title/Abstract]) OR "metabolic syndrome"[Title/Abstract]) OR "Metabolic"[Title/Abstract]) OR "cholesterol, hdl"[MeSH Terms]) OR "triglycerides"[MeSH Terms]) OR (("blood pressure"[MeSH Terms] OR "blood pressure determination"[MeSH Terms]) OR "arterial pressure"[MeSH Terms])) OR "metabolic syndrome"[MeSH Terms]) OR "waist circumference"[MeSH Terms]) OR "obesity, abdominal"[MeSH Terms])) OR "body mass index"[MeSH Terms]) AND ("predict*"[Title/Abstract] OR "predictive value of tests"[MeSH Terms])) OR "scor*"[Title/Abstract]) OR "observ*"[Title/Abstract]) OR "observer variation"[MeSH Terms]) OR "prognos*"[Title/Abstract]) AND (((((("GDM"[Title/Abstract] OR ("Gestational"[Title/Abstract] AND "Diabetes"[Title/Abstract])) OR (("Pregnancy"[Title/Abstract] AND "induced"[Title/Abstract]) AND "Diabetes"[Title/Abstract])) OR (("Pregnancy"[Title/Abstract] AND "related"[Title/Abstract]) AND "Diabetes"[Title/Abstract])) OR (("Gestational"[Title/Abstract] AND "Metabolic"[Title/Abstract]) AND "disorder"[Title/Abstract])) OR (("Gestational"[Title/Abstract] AND "glucose"[Title/Abstract]) AND "intolerance"[Title/Abstract])) OR ("Pregnancy"[Title/Abstract] AND "Hyperglycaemia"[Title/Abstract]))

**CINAHL:**

( TI ( “pregnant women” or pregnan* or maternal or “first trimester” ) OR AB ( “pregnant women” or pregnan* or maternal or “first trimester” ) OR MH ( “pregnant women” or pregnan* or maternal or “first trimester” ) ) AND ( TI (cholesterol or glucose or body mass index or lipids or hdl OR high density lipoprotein cholesterol OR triglyceride OR triacylglycerol OR blood pressure OR waist circumference OR central obesity OR “metabolic syndrome” OR metabolic) OR AB (cholesterol or glucose or body mass index or lipids or hdl OR high density lipoprotein cholesterol OR triglyceride OR triacylglycerol OR blood pressure OR waist circumference OR central obesity OR “metabolic syndrome” OR metabolic) OR MH (cholesterol or glucose or body mass index or lipids or hdl OR high density lipoprotein cholesterol OR triglyceride OR triacylglycerol OR blood pressure OR waist circumference OR central obesity OR “metabolic syndrome” OR metabolic) ) AND ( (predict or predictors or predictive or prediction) OR score OR observation OR (prognosis or outcome or recovery or predictor) OR algorithm OR multivariate ) AND ( glucose or "gestational diabetes" or gdm or "gestational diabetes mellitus" or "diabetes in pregnancy" or "glucose intolerance" )

**EMBASE:**

('pregnant women'/exp OR 'pregnant women' OR pregnan* OR 'maternal'/exp OR maternal OR 'first trimester') AND (predict* OR predictive) AND (value OR tests OR scor* OR observ* OR observer) AND (variation OR prognos* OR calibration OR indices OR algorithm* OR multivar*) NOT diagnos* AND ('glucose intolerance' OR 'gestational diabetes' OR gdm OR 'gestational mellitus' OR 'diabetes in pregnancy') AND ('article'/it OR 'article in press'/it OR 'review'/it)

**COCHRANE DATABASE:**

## (“pregnant women” OR pregnan* OR maternal OR “first trimester”) in Title Abstract Keyword AND cholesterol or glucose or “body mass index” or lipids or hdl OR “high density lipoprotein cholesterol” OR triglyceride OR triacylglycerol OR “blood pressure” OR “waist circumference” OR “central obesity” OR “metabolic syndrome” OR metabolic in Title Abstract Keyword AND ((predict* OR predictive) AND (value OR tests OR scor* OR observ* OR observer) AND (variation OR prognos* OR calibration OR indices OR algorithm* OR multivar*)) NOT diagnos* in Title Abstract Keyword AND “glucose intolerance” OR “gestational diabetes” OR gdm OR “gestational mellitus” OR “diabetes in pregnancy” in Title Abstract Keyword AND cholesterol or glucose or “body mass index” or lipids or hdl OR “high density lipoprotein cholesterol” OR triglyceride OR triacylglycerol OR “blood pressure” OR “waist circumference” OR “central obesity” OR “metabolic syndrome” OR metabolic in Title Abstract Keyword in Title Abstract Keyword - (Word variations have been searched)
