# Supplementary material for: Maternal metabolic factors and the association with gestational diabetes: A systematic review and meta‐analysis
Source: Diabetes Metab Res Rev. 2022 Apr 25;38(5):e3532. doi: 10.1002/dmrr.3532 (PMC9540632; doi:10.1002/dmrr.3532)
Supplement: Supplementary file 2 — Supplementary Material S2 [file DMRR-38-e3532-s001.docx]

**Supporting Information: Risk of bias for each of the included studies, using QUIPS risk of bias**

| **Author, Country** | **Study participation** | **Study attrition** | **Prognostic factor measurement** | **Outcome measurement** | **Adjustment for other prognostic factors** | **Statistical analysis and reporting** | **OVERALL RISK OF BIAS** |
| --- | --- | --- | --- | --- | --- | --- | --- |
| Madhavan 2008 | High | High | Low | Moderate | High | High | High |
| Cozzolino 2017 | Moderate | Low | Moderate | Low | High | High | High |
| Hinkle 2018 | Moderate | Moderate | Low | Low | Low | Low | Moderate |
| Zhu 2020 | Low | Low | Low | Low | Moderate | Moderate | Moderate |
| Godwin 1999 | Moderate | Low | Moderate | Low | Moderate | Moderate | Moderate |
| Grieger 2018 | Low | Low | Low | Moderate | Low | Low | Low |
| Doi 2020 | Low | Low | High | High | High | High | High |
| Yachi 2011 | Low | Low | Low | Low | Low | Low | Low |
| Iyoke 2013 | Low | Low | Low | Moderate | High | High | High |
| Schrauwers 2009 | Low | Low | Low | Moderate | High | High | High |
| Migda 2016 | Low | Low | Low | Moderate | High | Moderate | Moderate |
| Kouhkan 2018 | Low | Low | Low | Low | Low | Low | Low |
| Phaloprakarn 2009 | Moderate | Low | Low | Low | High | Moderate | Moderate |
| Sweeting 2017 | Low | Low | Low | Low | High | Moderate | Moderate |
| Gur 2014 | High | Moderate | High | Low | High | Moderate | High |
| Lei 2016 | Low | Low | Low | Low | Moderate | Moderate | Moderate |
| Zhu 2019 | Low | Low | Low | Low | Low | Low | Low |
| Zhu 2013 | Low | Moderate | Low | Low | Low | Moderate | Low |
| Zhang 2019 | Low | Low | Low | Low | Low | Moderate | Low |
| Magann 2013 | Moderate | Low | Low | Moderate | Low | Low | Low |
| Zhao 2014 | Moderate | Low | Low | High | Moderate | High | High |
| Simko 2019 | Low | Low | Low | Low | Moderate | Low | Low |
| O'Malley 2020 | Moderate | Moderate | Moderate | Moderate | Moderate | Moderate | Moderate |
| Wang 2017 | Low | Low | Low | Low | Low | Moderate | Low |
| El-Gilany 2010 | Moderate | Moderate | Low | High | High | High | High |
| Wen-Yuan 2016 | Low | Low | Low | Low | Low | High | Low |
| Denison 2014 | Low | Moderate | Low | Moderate | Low | Moderate | Moderate |
| Han 2018 | Low | Low | Low | Low | Low | Low | Low |
| Pazhohan 2019 | Low | Low | High | Low | Moderate | Moderate | Moderate |
| Syngelaki 2011 | Moderate | Low | Low | Low | Low | Low | Low |
| Sánchez-Vera 2007 | Moderate | High | High | Moderate | Moderate | Moderate | Moderate |
| Falcone 2019 | Moderate | High | Low | Low | Moderate | Moderate | Moderate |
| Sesmilo 2019 | Moderate | Moderate | High | Low | Moderate | Moderate | Moderate |
| Amylidi 2016 | Moderate | Moderate | Moderate | Low | High | High | High |
| Vellamkondu 2017 | High | High | Moderate | High | High | High | High |
| Wang 2013 | Moderate | Moderate | Moderate | Low | Moderate | Moderate | Moderate |
| Knight-Agarwal 2016 | Moderate | Moderate | Moderate | High | Moderate | Moderate | Moderate |
| Collier 2017 | Moderate | Moderate | Moderate | High | Moderate | Moderate | Moderate |
| Savvidou 2010 | Moderate | Moderate | Low | Low | Low | Low | Low |
| Kansu-Celik 2019 | Moderate | Low | Low | Low | High | High | High |
| Farah 2012 | High | High | High | High | High | High | High |
| Bao 2018 | Low | Low | Low | Low | Low | Low | Low |
| Odsæter 2015 | Low | Low | Low | Low | High | High | High |
| Yang 2019 | Low | Moderate | Moderate | High | Low | Low | Moderate |
| Sreedevi 2012 | High | High | Moderate | High | High | High | High |
| Zheng 2019 | Low | Moderate | Moderate | Low | High | High | High |
| Sánchez-García 2020 | Low | Low | Low | Low | Low | Low | Low |
| Hashemi-Nazari 2020 | High | Moderate | Moderate | Moderate | Low | Low | Moderate |
| Wani 2020 | Moderate | High | Low | Low | Low | Low | Moderate |
| Berggren 2017 | Low | Moderate | Moderate | Low | High | High | High |
| Grewal 2012 | Low | High | Low | Low | High | High | High |
| Ogonowski 2007 | Low | Low | Moderate | Low | High | High | High |
| Arbib 2019 | Low | Moderate | Low | Low | High | High | High |
| Teede 2011 | Low | High | Low | Low | Low | Low | Low |
| Punnose 2020 | Low | Low | Moderate | Low | Low | Low | Low |
| Berggren 2015 | Moderate | Moderate | Low | Low | Low | Low | Low |
| Li 2016 | High | High | Low | Low | Low | Low | Moderate |
| Riskin-Mashiah 2010 | Moderate | High | Moderate | Low | Moderate | Moderate | Moderate |
| Raja 2012 | Moderate | Moderate | Low | High | Low | Low | Moderate |
| Gabbay-Benziv 2015 | Low | Moderate | Moderate | Low | Moderate | Moderate | Moderate |
| Basraon 2016 | Low | Low | Low | Moderate | Low | Low | Low |
| Alptehkin 2016 | Moderate | Low | Low | Low | High | High | High |
| Li 2019 | Low | Low | Low | Moderate | High | High | High |
| Wolfe 1991 | High | High | Low | Low | High | High | High |
| Nanda 2011 | High | Low | High | Low | Low | Moderate | Moderate |
| Kumru 2016 | High | High | Low | Low | Low | Low | Low |
| Hancerliogullari 2020 | Moderate | Low | Low | Low | High | High | High |
| Ozgu-Erdinc 2019 | Moderate | Low | High | Low | High | High | High |
| Gao 2020 | Moderate | Low | Moderate | Low | High | High | High |
| Liu 2020 | Moderate | Moderate | Low | Low | High | High | High |
| Meek 202 | Moderate | Low | High | Moderate | High | High | High |
| Guo 2020 | Moderate | Moderate | High | Low | Low | Low | Moderate |
| Wang 2016 | Low | Low | Low | Low | High | High | High |
| Al-Shafei 2021 | Moderate | Low | High | Low | High | High | High |
| Zhang 2020 | Low | Low | High | Low | High | High | High |
| Tenenbaum-Gavish 2020 | Moderate | Low | Low | Low | High | High | High |
| Leng 2015 | Moderate | Low | Low | Low | Low | Low | Low |
| Schneider 2021 | Moderate | Low | Low | Low | Low | Low | Low |
