# Supplementary material for: Maternal metabolic factors and the association with gestational diabetes: A systematic review and meta‐analysis
Source: Diabetes Metab Res Rev. 2022 Apr 25;38(5):e3532. doi: 10.1002/dmrr.3532 (PMC9540632; doi:10.1002/dmrr.3532)
Supplement: Supplementary file 3 — Supplementary Material S3 [file DMRR-38-e3532-s003.docx]

**Supporting Information: Figures on mean (SD) for metabolic factors and GDM**


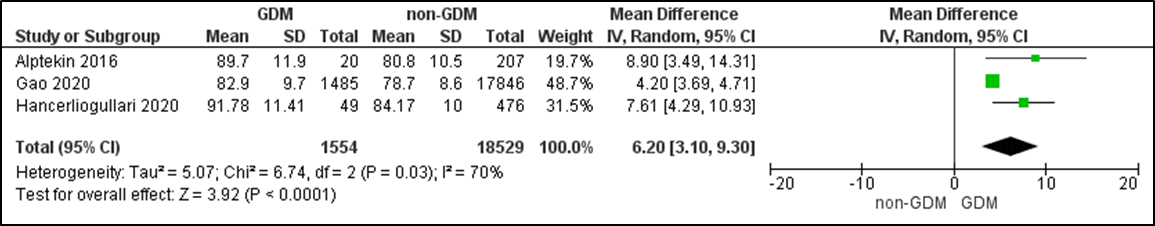


**Figure 1: Mean difference of early pregnancy waist circumference between women with and without gestational diabetes.** Values are mean (SD). For overall effect, a p-value <0.05 was considered significant.


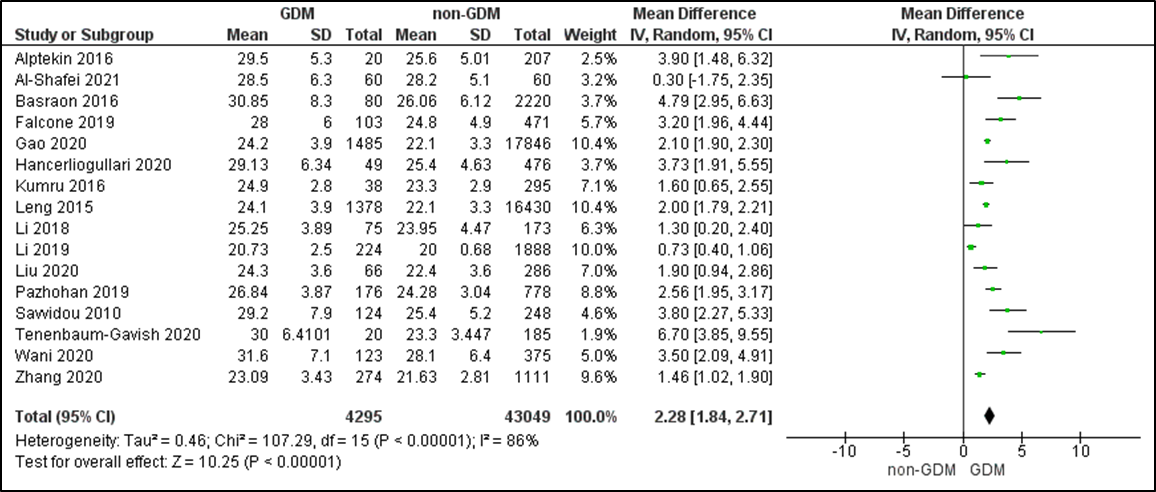


**Figure 2: Mean difference of early pregnancy BMI between women with and without gestational diabetes.** Values are mean (SD). For overall effect, a p-value <0.05 was considered significant.


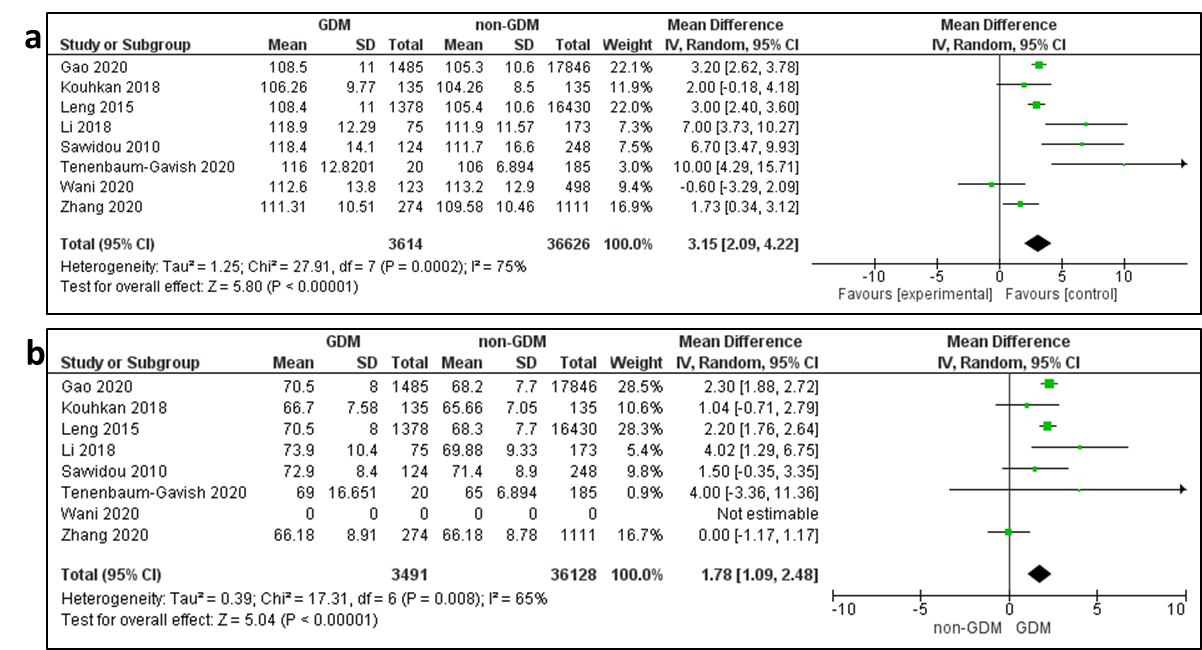


**Figure 3: Mean difference of early pregnancy blood pressure between women with and without gestational diabetes.** Values are mean (SD). a) systolic and b) diastolic blood pressure. For overall effect, a p-value <0.05 was considered significant.


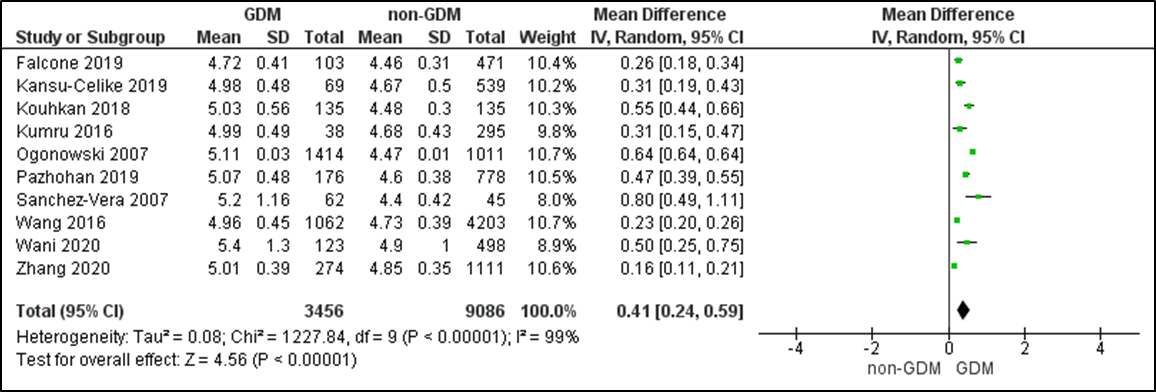


**Figure 4: Mean difference of early pregnancy fasting plasma glucose between women with and without gestational diabetes.** Values are mean (SD). For overall effect, a p-value <0.05 was considered significant.


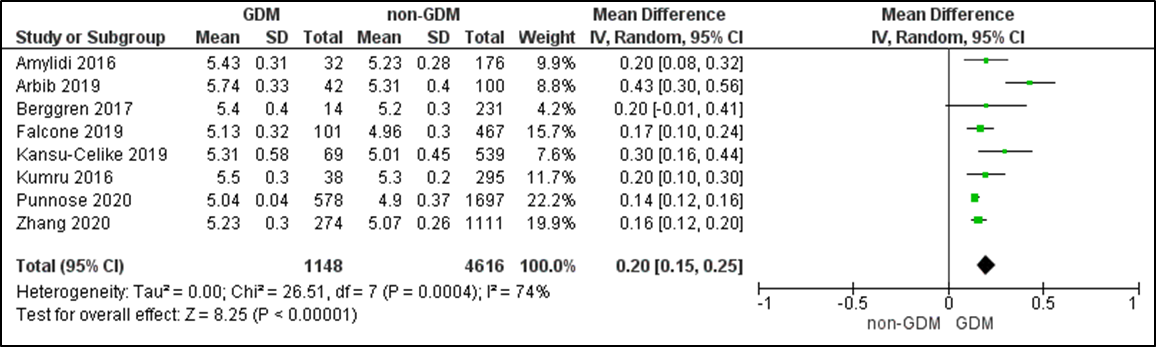


**Figure 5: Mean difference of early pregnancy glycosylated haemoglobin (HbA1c) between women with and without gestational diabetes.** Values are mean (SD). For overall effect, a p-value <0.05 was considered significant.


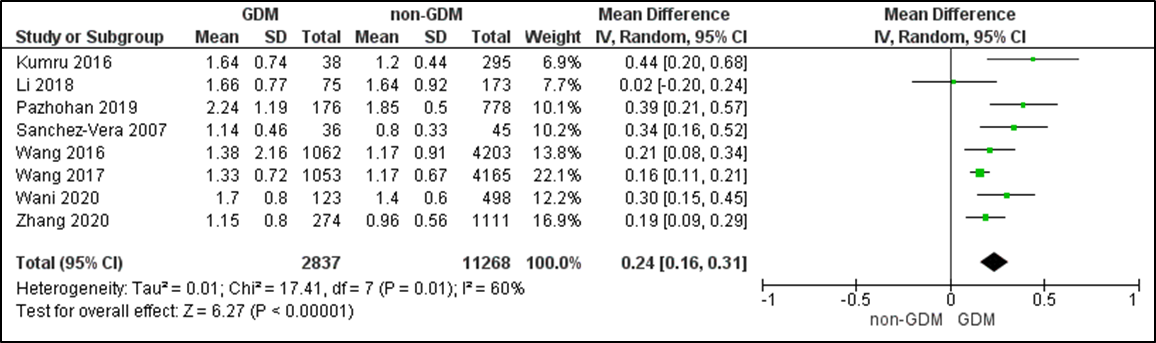


**Figure 6: Mean difference of early pregnancy triglycerides (TG) between women with and without gestational diabetes.** Values are mean (SD). For overall effect, a p-value <0.05 was considered significant.


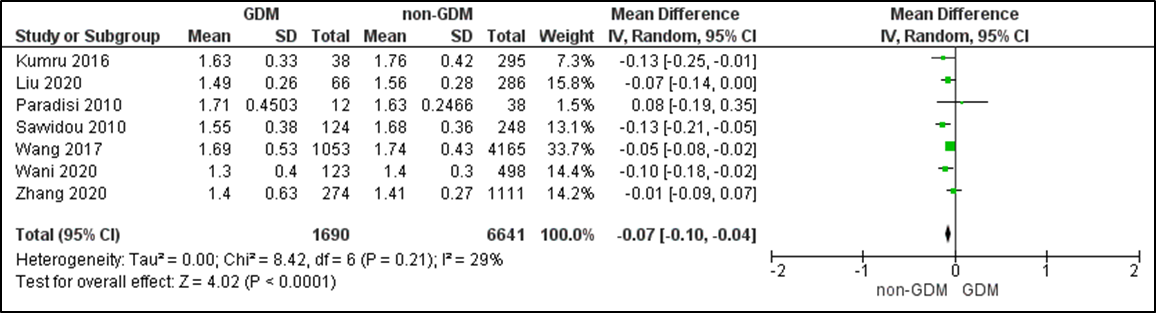


**Figure 7: Mean difference of early pregnancy high-density lipoprotein cholesterol (HDL-C) between women with and without gestational diabetes.** Values are mean (SD). For overall effect, a p-value <0.05 was considered significant.

**Supporting Information: Figures assessing between-study heterogeneity for metabolic factors and GDM**


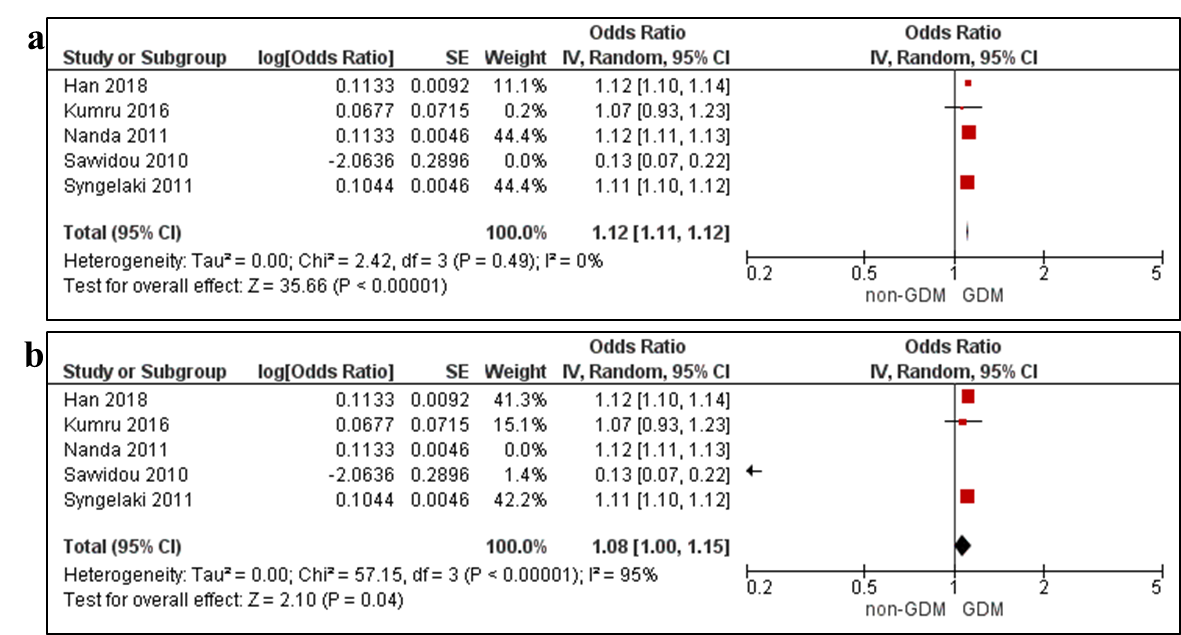


**Figure 8: Assessment of between-study heterogeneity in the meta-analysis of early pregnancy BMI and adjusted odds of gestational diabetes.** a) by eliminating the study with a different direction of effect, b) by removing the study with high risk of bias.


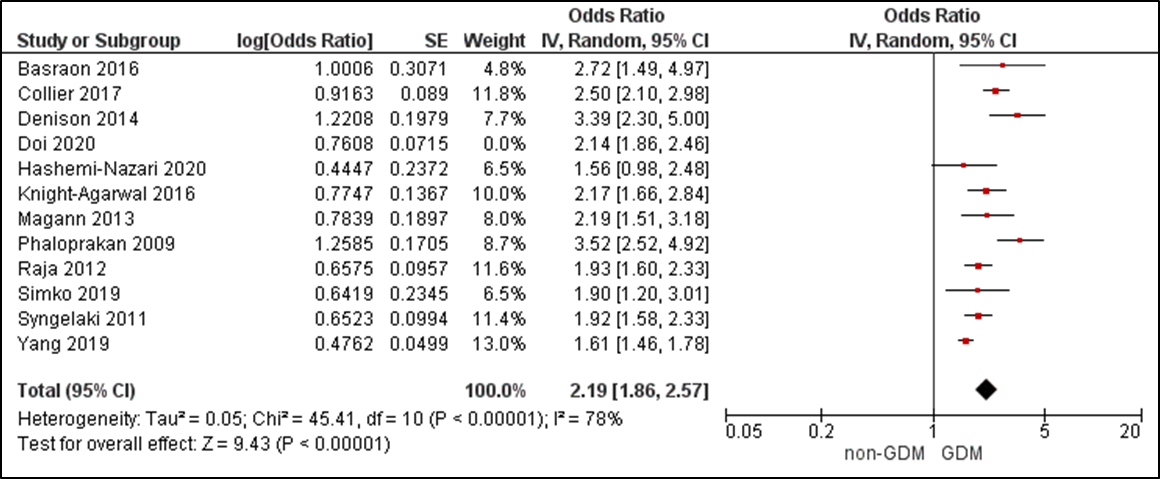


**Figure 9: Assessment of between-study heterogeneity in the meta-analysis of early pregnancy overweight and adjusted odds of gestational diabetes** by removing the study with high risk of bias.

**
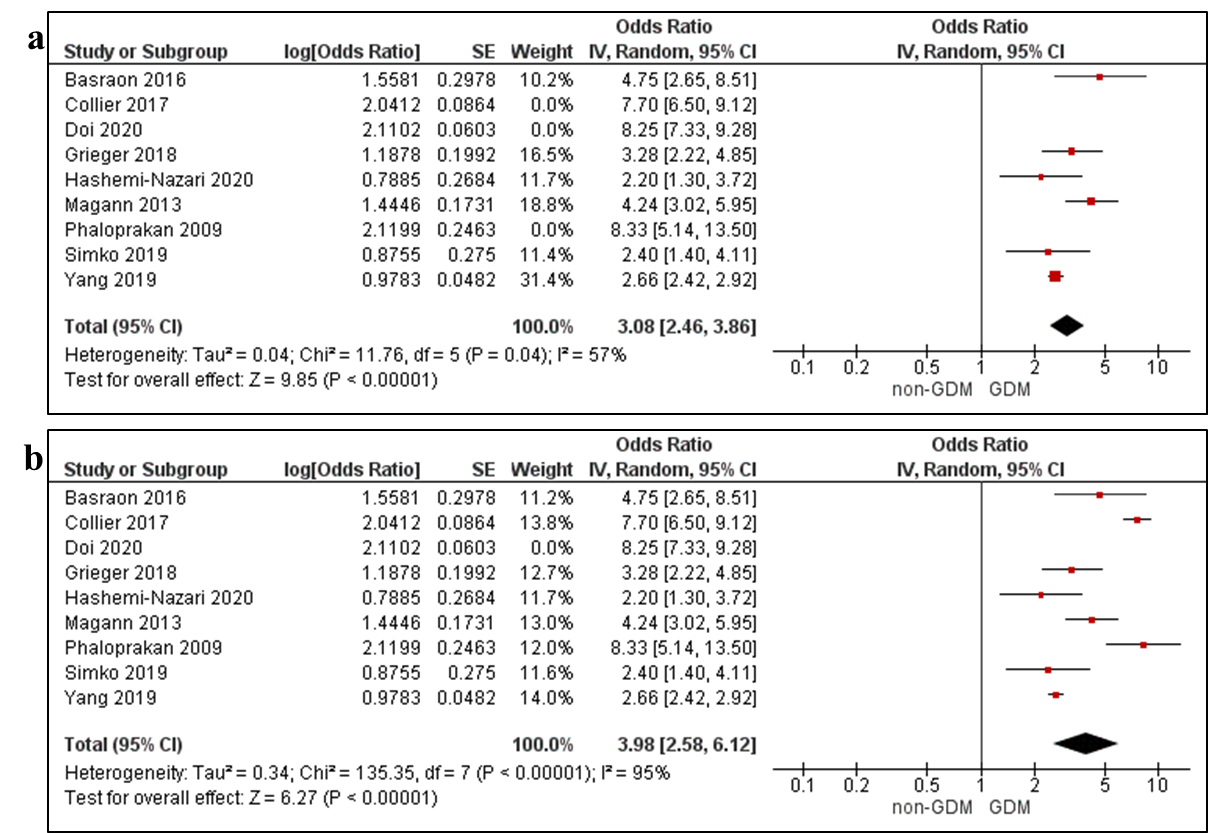
**

**Figure 10: Assessment of between-study heterogeneity in the meta-analysis of early pregnancy obesity and adjusted odds of gestational diabetes.** a) by eliminating three studies with a large difference in effect estimate (removing each study, I^2^ ranged from 96% -98%). b) by removing the study with high risk of bias.


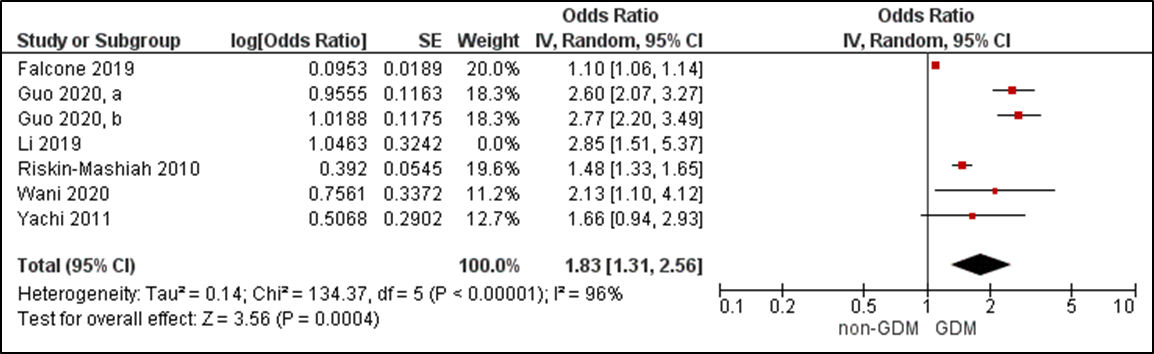


**Figure 11: Assessment of between-study heterogeneity in the meta-analysis of early pregnancy fasting plasma glucose for adjusted odds of gestational diabetes** by removing the study with high risk of bias.


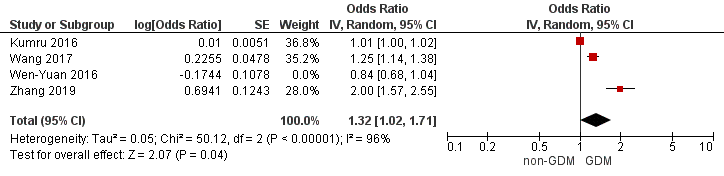


**Figure 12: Assessment of between-study heterogeneity in the meta-analysis of early pregnancy triglycerides for adjusted odds of gestational diabetes** by eliminating the study with a different direction of effect.
