# Supplementary material for: Maternal metabolic factors and the association with gestational diabetes: A systematic review and meta‐analysis
Source: Diabetes Metab Res Rev. 2022 Apr 25;38(5):e3532. doi: 10.1002/dmrr.3532 (PMC9540632; doi:10.1002/dmrr.3532)
Supplement: Supplementary file 4 — Figure S1 [file DMRR-38-e3532-s002.docx]

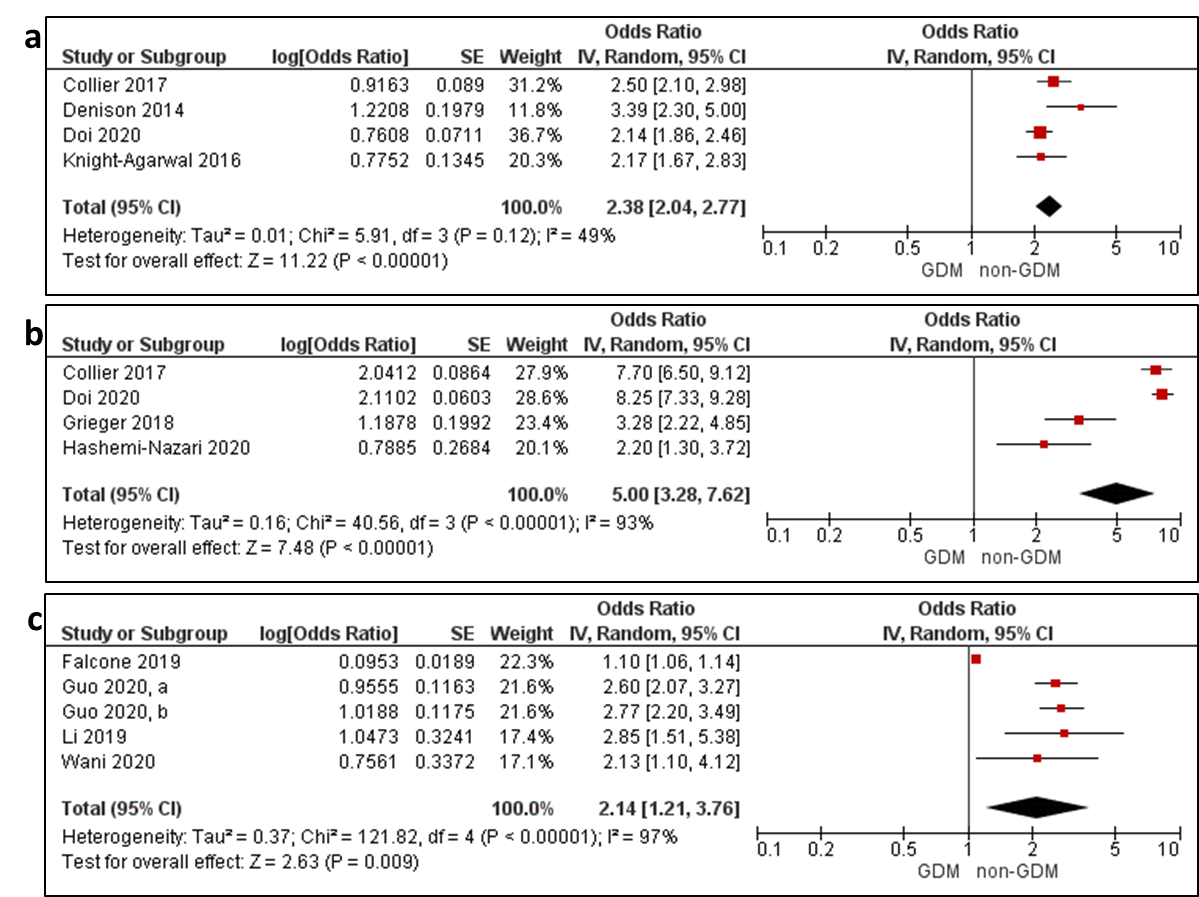


**Supplementary Figure 13: Meta-analysis of early pregnancy a) overweight, b) obesity and c) fasting plasma glucose and odds of gestational diabetes (GDM) based on the respective 2013 WHO criteria [1], 2013 WHO/ADA [2] criteria, and 2010 IADPSG [3]/ADA diagnostic criteria for GDM.** Values are odds ratios with 95% CIs adjusted for maternal age and BMI.

1. World Health Organization., *Definition, Diagnosis and Classification of Diabetes Mellitus and its Complications. 1999. Part 1: Diagnosis and Classification of Diabetes Mellitus.* [*https://apps.who.int/iris/handle/10665/66040*](https://apps.who.int/iris/handle/10665/66040).

2. American Diabetes, A., *Standards of medical care in diabetes--2010.* Diabetes Care, 2010. **33 Suppl 1**: p. S11-61.

3. International Association of Diabetes and Pregnancy Study Groups., *Recommendations on the Diagnosis and Classification of Hyperglycemia in Pregnancy. Diabetes Care. 2010;33(3):676. doi:10.2337/dc09-1848*.
